# IAPs in cancers: molecular mechanisms, clinical prognostic value, and translational therapeutic potential

**DOI:** 10.1186/s12967-025-07640-7

**Published:** 2026-01-08

**Authors:** Zhisheng Teng, Liyun Teng, Jing Xie

**Affiliations:** 1 Department of Head and Neck Surgery, Zhejiang Cancer Hospital, Hangzhou Institute of Medicine (HIM), Chinese Academy of Sciences, Hangzhou, China; 2 Department of Obstetrics and Gynecology, The Chinese Medicine Hospital of Yongjia County, Wenzhou, China; 3 Department of Stomatology, The First Affiliated Hospital of Wenzhou Medical University, Wenzhou, China

**Keywords:** Inhibitor of apoptosis proteins (IAPs), Ubiquitination, Cell death, Innate and adaptive immunity, IAPs inhibitors

## Abstract

**Background:**

Conventional therapies remain the primary approach for most cancers but typically achieve only modest improvements in prognosis. With the rapid advances in molecular biology and multi-omics technologies, targeted therapy has become the first choice for treating advanced cancer. Among these, inhibitor of apoptosis proteins (IAPs) has emerged as critical regulators in both experimental and clinical studies.

**Main body:**

Nevertheless, several challenges persist, including tumor heterogeneity across cancer types, adverse effects associated with IAPs inhibitors (such as cytokine release and inflammation), and the lack of validated biomarkers for patient selection. With the development of artificial intelligence and precision medicine, IAP-targeted therapy, especially in combination with other therapies, has shown favorable clinical application potential.

**Conclusions:**

This review systematically summarizes the structural domains, molecular functions, biological processes, clinical relevance, and advances in drug development and translational applications of IAPs, aiming to refine therapeutic strategies and facilitate clinical translation.

## Introduction

Cancer remains a major global health challenge due to its biological heterogeneity, complex etiologies, and diverse clinical manifestations [1] Despite advances in surgery, radiotherapy, and chemotherapy, treatment efficacy is frequently limited by multidrug resistance, tumor recurrence, and the high proportion of patients who present with unresectable or metastatic disease [2]. Achieving selective tumor cell eradication while minimizing systemic toxicity therefore remains a central but unresolved clinical objective [3].

The rapid development of molecular oncology has enabled the identification of actionable biomarkers and therapeutic targets, promoting a shift toward personalized and mechanism-driven cancer therapies [4]. Among these targets, the Inhibitor of Apoptosis Proteins (IAPs), also referred to as BIR-repeat containing proteins (BIRCs), have emerged as key regulators.

Beyond their canonical role in suppressing apoptosis, IAPs exhibit E3 ubiquitin ligase activity [5], orchestrate multiple forms of cell death including necroptosis and pyroptosis [6], and participate in the modulation of innate and adaptive immune responses [7]. Dysregulated IAP expression is closely associated with tumor progression, therapy resistance, and unfavorable clinical outcomes across a range of malignancies, highlighting their translational relevance as therapeutic targets [8].

**Fig. 1:**
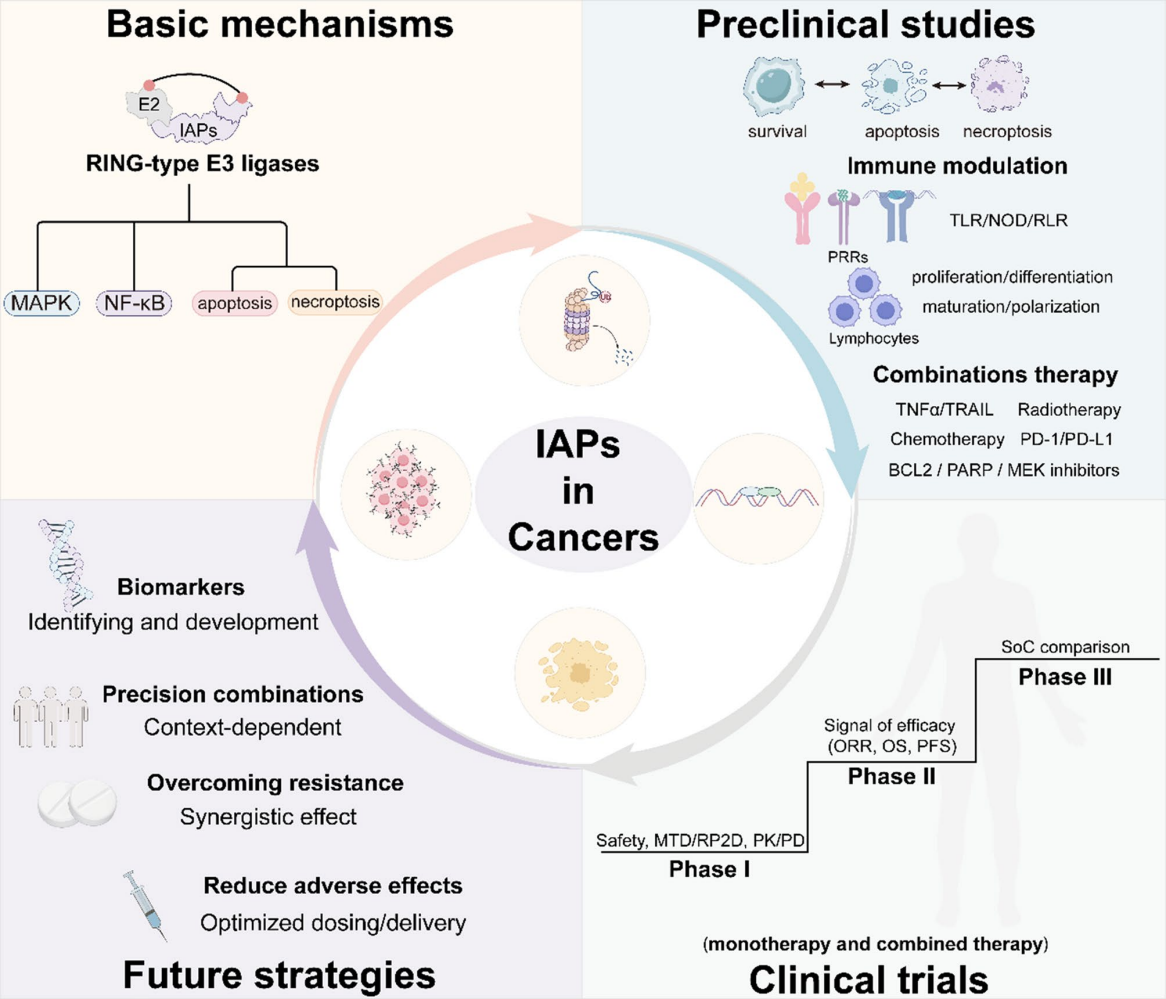
Translational roadmap from basic mechanisms to clinical progression and future strategies for IAP-targeted therapies. The upper left panel gives a brief overview of IAPs’ main functions, such as their RING-type E3 ligase activity and their roles in regulatory MAPK and NF-κB signaling, apoptosis, and necroptosis. The upper right panel presents key findings from preclinical studies, including their involvement in cell survival and cell death pathways, immune modulation through pattern recognition receptors (PRRs), and effects on lymphocyte proliferation, differentiation, maturation, and polarization. This section also outlines combination strategies under investigation, such as chemotherapy, radiotherapy, TNFα/TRAIL, PD-1/PD-L1 blockade, and targeted inhibitors of BCL2, PARP, and MEK. The lower right panel depicts the stepwise clinical progression of IAP-targeted therapies, from Phase I trials assessing safety, MTD/RP2D, and PK/PD, to Phase II studies evaluating preliminary efficacy (ORR, OS, PFS), and Phase III trials comparing outcomes with standard-of-care regimens. The lower left panel highlights future directions, including biomarker discovery, precision combinations tailored to tumor context, strategies to overcome therapeutic resistance, and approaches to reduce adverse effects through optimized dosing and delivery systems

This review provides a comprehensive overview of the structural domains, molecular functions, biological processes, clinical relevance, drug development and translational applications. By integrating structural, molecular, biological, and translational insights, this article aims to clarify the multifaceted roles of IAPs in cancer and to highlight emerging opportunities and challenges in optimizing IAP-related therapies (Fig. 1).

## Structural domains of IAPs

**Fig. 2:**
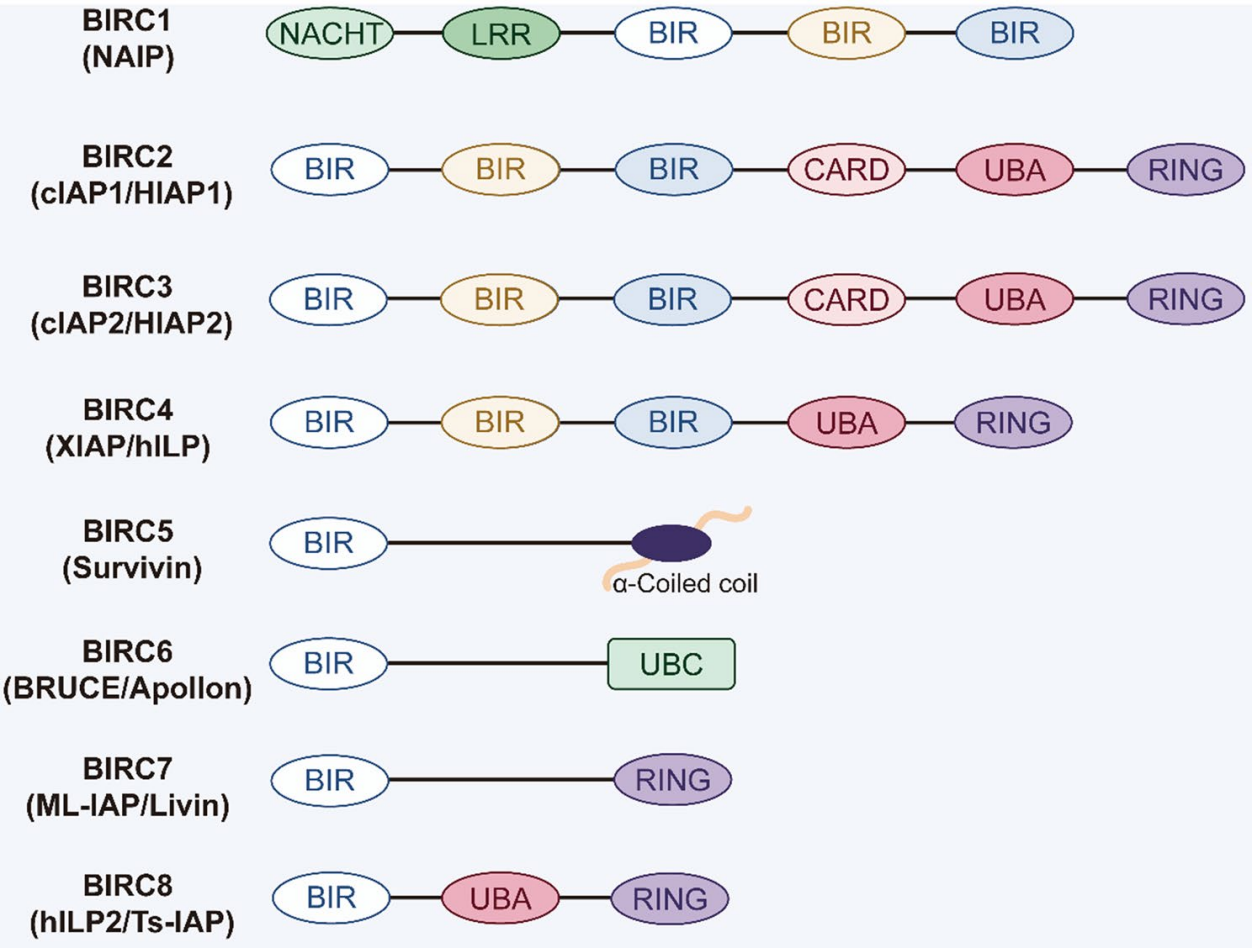
Domains of the inhibitor of apoptosis proteins family. BIR: Baculovirus IAP Repeat; RING: Really Interesting New Gene; UBA: Ubiquitin-Associated; CARD: Caspase Recruitment; UBC: Ubiquitin-Conjugating; NACHT Domain: Naip-C2TA-HetE-Tep1 Nucleotide-Binding and Oligomerization; LRR: Leucine-Rich Repeat

In 1993, Miller and colleagues identified the first inhibitor of apoptosis protein (IAP) from a baculovirus strain, Op-IAP, which suppresses apoptosis to support viral replication [9]. Subsequent studies revealed the presence of IAP homologs in both vertebrates and invertebrates, leading to the characterization of eight mammalian IAPs, also termed BIRCs (Baculoviral IAP Repeat-Containing proteins) [10]. These include BIRC1 (neuronal apoptosis inhibitory protein/NAIP), BIRC2 (cellular IAP1/cIAP1/HIAP1), BIRC3 (cellular IAP2/cIAP2/HIAP2), BIRC4 (X-linked IAP/XIAP/hILP), BIRC5 (Survivin), BIRC6 (BIR-containing ubiquitin-conjugating enzyme/BRUCE/Apollon), BIRC7 (melanoma IAP/ML-IAP/Livin), and BIRC8 (IAP-like protein 2/hILP2/Ts-IAP) [11, 12]. All IAPs are characterized by a zinc-binding Baculovirus IAP Repeat (BIR) domain of ~70–80 amino acids [13]. Additional domains, including RING, UBA, CARD, UBC, NACHT, LRR, and coiled-coil domain, endow individual IAPs with distinct structural and functional properties (Fig. 2). A detailed description of each domain is provided here to establish the structural basis for understanding their molecular functions and regulatory mechanisms.

BIR Domain (Baculovirus IAP Repeat Domain): The BIR domain is a zinc-binding structural motif primarily mediating binding to caspases and other signaling proteins. Based on the presence of a peptide-binding groove, BIR domains are classified as type I (without a groove or with a shallow pocket) or type II, which contain an IAP-binding motif (IBM) at the N-terminus [14]. Notably, BIR domains of BIRC1, 2, 3, 4, 7, and 8 interact directly with caspase, whereas BIRC5 and 6, as well as those in yeast and Caenorhabditis elegans, execute non-apoptotic roles in cytokinesis and spindle assembly [15]. Structurally, BIRC1-4 possess three tandem BIR domains, while BIRC5-8 contain single BIR motifs [16].

RING Domain (Really Interesting New Gene Domain): The RING domain is a cysteine-rich motif composed of seven conserved cysteines and one histidine residue that coordinate two zinc ions and confer E3 ubiquitin ligase activity [17]. This domain enables IAPs to recruit E1 and E2 enzymes and catalyze the ubiquitination of substrate proteins, thereby regulating their degradation, stabilization, and signal transduction [18]. Notably, BIRC2, 3, 4, 7, and 8 each harbor a single RING domain [19].

UBA Domain (Ubiquitin-Associated Domain): The UBA domain, located between the BIR and RING domains, binds mono- and polyubiquitin chains, facilitating protein complex assembly and diverse signaling pathways [20]. BIRC2, 3, 4, and 8 each encode a single UBA domain [21].

CARD Domain (Caspase Recruitment Domain): The CARD domain, found in BIRC2 and BIRC3, inhibits RING domain dimerization and limits E3 ubiquitin ligase activity through direct interaction with E2 enzymes [22]. BIRC2 and BIRC3 harbor a single CARD domain that spatially constrains RING domain functionality through its conserved α-helical-α-helical interface [23].

UBC Domain (Ubiquitin-Conjugating Domain): BIRC6 lacks both RING and UBA domains but uniquely encodes a UBC domain that confers dual E2/E3 ubiquitin ligase activity. This domain enables BIRC6 to ubiquitinate and promote proteasomal degradation of proapoptotic proteins [24].

NACHT Domain (Naip-C2TA-HetE-Tep1 Nucleotide-Binding and Oligomerization Domain): BIRC1 (NAIP) contains a NACHT domain that exerts dual regulatory functions in blocking apoptotic pathways through caspase inhibition and promoting transcriptional activation of major histocompatibility complex class II (MHC II) [25].

LRR Domain (Leucine-Rich Repeat Domain): At the C-terminus, BIRC1 (NAIP) also contains an LRR domain, a critical component of the NOD-like receptor (NLR) family, which actively participates in host-pathogen recognition and innate immune signaling pathways [26].

Coiled Coil Domain: BIRC5 (Survivin) features a C-terminal coiled-coil domain, an amphipathic α-helical structure displayed in microtubule-associated proteins [21].

## Molecular functions of IAPs

The ubiquitin–proteasome system (UPS) governs selective protein degradation and signaling by attaching ubiquitin to substrates through a sequential E1–E2–E3 enzymatic cascade, with DUBs and the 26S proteasome maintaining dynamic ubiquitin turnover and proteostasis [27]. Ubiquitin, a conserved 76-amino-acid polypeptide containing an N-terminal methionine (M1) and seven lysine residues (K6, K11, K27, K29, K33, K48, and K63), supports diverse linkage types whose topologies dictate outcomes such as K48-linked degradation or K63-linked signaling [28].

The E1 activating enzymes, a small family of ATP-dependent initiators of the ubiquitin and its thioester linkage to a catalytic cysteine, prime it for transfer to E2 conjugating enzymes [29]. E2 enzymes, defined by a 150–200 amino acid UBC domain, receive ubiquitin from E1s and cooperate with E3 ligases to determine substrate specificity and chain architecture [30, 31]. E3 ligases catalyze the final transfer of ubiquitin to targets and are classified into single-subunit families such as HECT, RING, U-box, and N-recognin as well as multisubunit complexes such as Cullin-RING and APC/C [32, 33]. DUBs, including cysteine proteases (UCH, USP, OTU, MJD, MINDY, MCPIP, and ZUFSP families) and metalloproteases (JAMM/MPN+ enzymes), cleave ubiquitin chains or release free ubiquitin to maintain signaling fidelity [34, 35]. The 26S proteasome, composed of a 19S regulatory particle (RP) and a 20S core particle (CP), recognizes, deubiquitinates, unfolds, and degrades target proteins to maintain protein homeostasis [36–39]. In this hierarchical cascade, E1 enzymes activate ubiquitin, E2s form thioester intermediates, and E3 ligases attach ubiquitin to substrate lysines. This directs proteins toward either proteasomal degradation or regulatory signaling. This tightly controlled process ensures proteostasis, stress adaptation, and signal transduction across cellular contexts [40–43].

Within this system, IAPs constitute a unique subclass of RING-type E3 ubiquitin ligases that link ubiquitination directly to cell regulation. The C-terminal RING domain of cIAP1, cIAP2, XIAP, Livin, and hILP2 mediates ubiquitin transfer from E2 enzymes to specific substrates, forming K6-, K11-, K27-, K48-, and K63-linked chains independently of intermediate E3 enzymes [44]. In addition, the UBC domain of BRUCE/Apollon endows it with both E2 and E3 ubiquitin enzyme activities [24]. cIAP1/2 act as master regulators of NF-κB signaling—their K63-linked ubiquitination of RIPK1 promotes canonical NF-κB activation, whereas K48-linked ubiquitination and degradation of NIK inhibit the non-canonical pathway, thus maintaining inflammatory balance and preventing spontaneous apoptosis [45, 46]. Additionally, cIAP1/2 also modulates apoptosis, cytoskeleton regulation, inflammation, and transcription [47]. XIAP exhibits broad E3 activity toward caspase-3, −7, −8, and −9, as well as regulatory proteins including Smac, PTEN, Chk1, RhoA, Rac1, and RIPK2, regulating apoptotic, cell-cycle, autophagy, DNA damage responses, copper homeostasis, and inflammatory signaling [48]. Livin selectively ubiquitinates Smac for proteasomal degradation through BIR-domain recognition and RING-domain catalysis [16]. HILP2 mediates the ubiquitination of itself and target proteins, including caspases and Smac. Through cooperation with the caspase activation and recruitment domain (CARD), it reduces apoptosis-related protein activity and inhibits apoptosis [49]. Distinctly, BRUCE/Apollon possesses both E2 and E3 ubiquitin enzyme activity via its internal UBC domain, functioning with UBA6 to ubiquitinate caspases-3, −7, −9, and LC3B, thereby linking apoptosis suppression to autophagy regulation [24, 50]. Notably, BRUCE/Apollon specifically ubiquitinates Kras4A (but not Kras4B), revealing a Hippo pathway regulatory mechanism in Drosophila [51].

In addition to their functions as E3 ubiquitin ligases, IAPs can directly inhibit caspases by blocking substrate access to their catalytic sites. XIAP is the only IAP that acts as a direct biochemical caspase inhibitor, suppressing caspase activity through physical binding. Caspase-3 and −7 inhibition occurs when the linker region between the BIR1 and BIR2 domains of XIAP occupies the catalytic site of these cysteine proteases, preventing substrate entry, while BIR2 simultaneously engages the IAP-binding motif (IBM) of the target protein. This dual interaction effectively docks and inhibits effector caspases. Caspase-9 is inhibited through a distinct mechanism in which the BIR3 domain binds its homodimerization interface, thereby interfering with the formation of the active catalytic pocket [52].

**Fig. 3:**
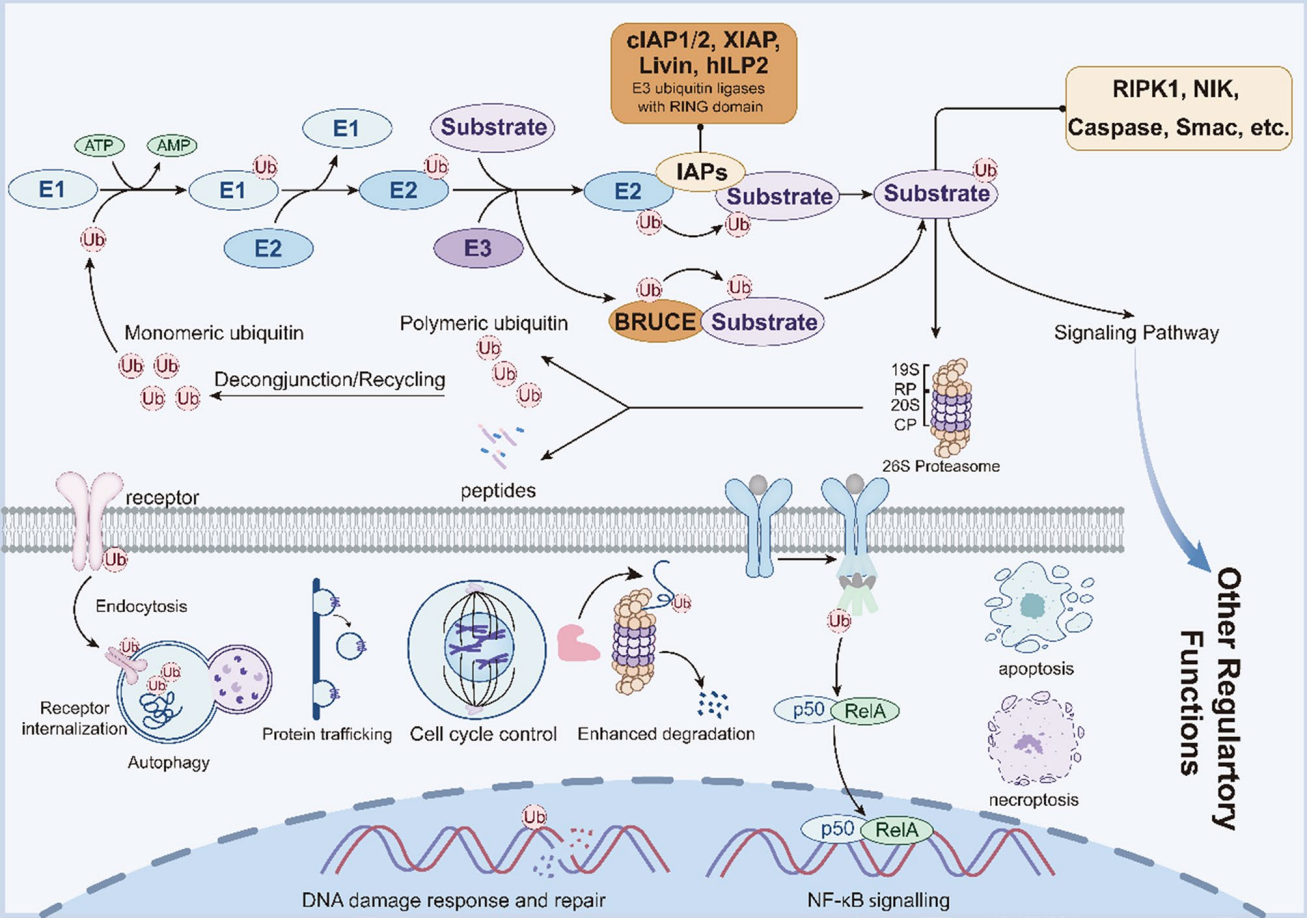
IAPs, functioning as E3 ubiquitin ligases, catalyze substrate ubiquitination within the ubiquitin-proteasome system (UPS), mediating either proteolytic degradation or other regulatory functions. E1 ubiquitin-activating enzymes create adenylated ubiquitin by hydrolyzing ATP. The catalytic cysteine residue of E2 ubiquitin-conjugating enzymes then receives this activated ubiquitin, creating an E2~ubiquitin thioester intermediate. E3 ubiquitin ligases recognize target proteins via distinct structural domains and catalyze the covalent attachment of ubiquitin from E2 enzymes to substrate lysine residues, thereby regulating either proteasome-mediated degradation or other regulatory functions such as regulation of apoptosis, necroptosis, NF-κB signaling, enhanced degradation, cell-cycle control, protein trafficking, DNA-damage response and repair, autophagy, etc. cIAP1, cIAP2, XIAP, Livin, and hILP2 exhibit intrinsic E3 ubiquitin ligase activity mediated by their C-terminal RING domains, enabling direct conjugation of polyubiquitin chains or monoubiquitin to target proteins without requiring intermediate E3 enzymes. BRUCE possesses both E2 and E3 ubiquitin enzyme activity via its internal UBC domain

Collectively, IAPs serve as multifunctional regulators that couple ubiquitin-dependent and ubiquitin-independent mechanisms to regulate biological function. As RING-type E3 ubiquitin ligases, they direct the ubiquitination of key signaling proteins—including RIPK1, components of the NF-κB pathway, and caspases—governing the balance between pro-survival, apoptotic, and necroptotic programs. Through these activities, IAPs shape tumor initiation, progression, and therapeutic response by integrating proteasomal degradation with signal transduction (Fig. 3). Dysregulated IAP-mediated ubiquitination contributes to malignant transformation and treatment resistance. A more comprehensive understanding of their E3 ligase–dependent networks and caspase-inhibitory functions will inform the development of refined IAP-targeted treatments and rational combination strategies in cancer therapy.

## Biological processes regulated by IAPs

IAPs participate in multiple biological processes that are essential for maintaining cellular and immune homeostasis. As ubiquitin E3 ligases, they integrate signals that influence both cell fate decisions and the activation and function of innate and adaptive immune responses. These interconnected roles position IAPs as central modulators of tissue homeostasis, host defense, and tumor biology. The following subsections outline two major biological processes regulated by IAPs: cell fate regulation and immune modulation.

### Cell fate regulation

**Fig. 4:**
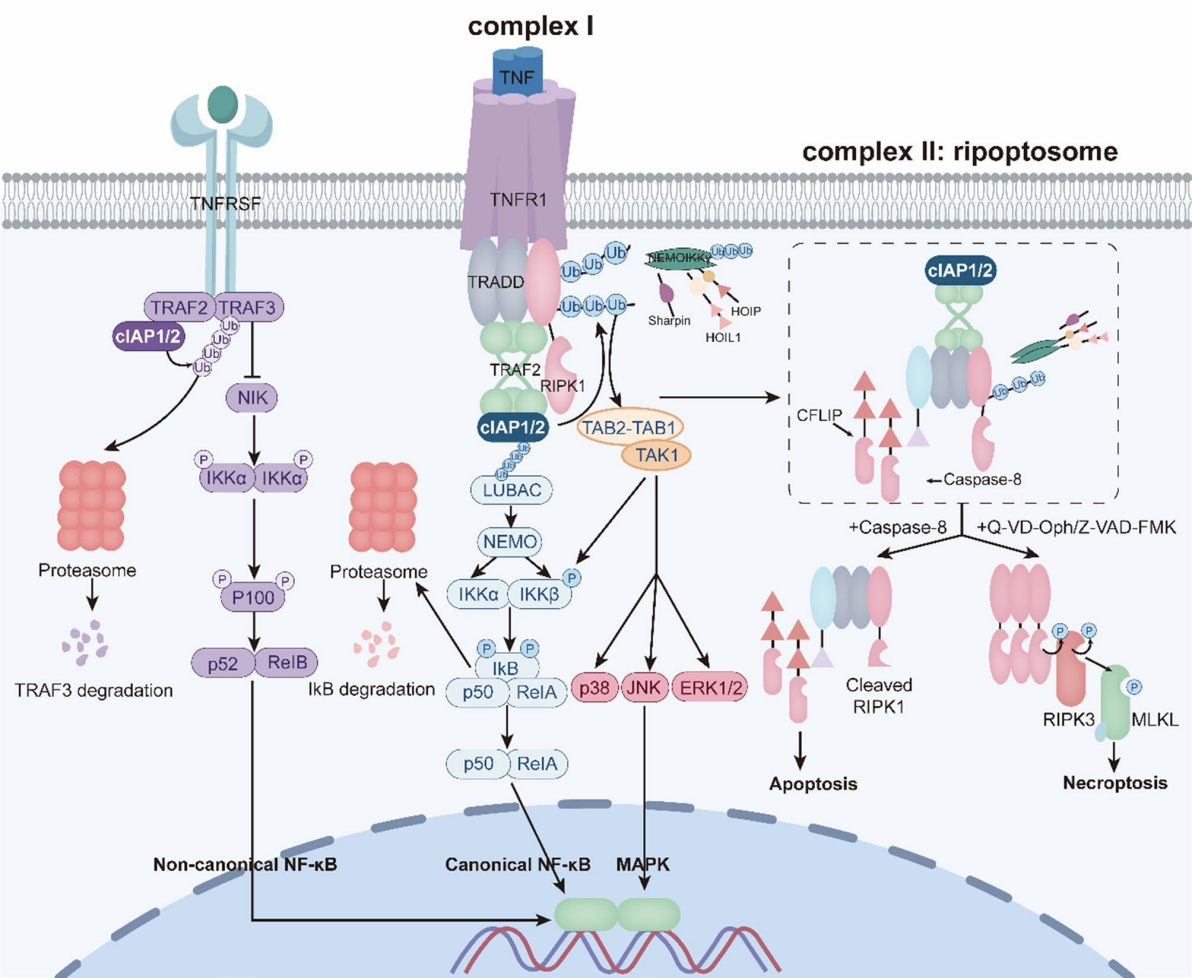
IAPs orchestrate cell fate decisions by balancing survival, apoptosis, and necroptosis. Upon TNF binding to TNFR1, complex I assembles at the membrane with TRADD, RIPK1, TRAF2, and cIAP1/2. cIAP1/2 recruit TAK1–TAB2/3 and LUBAC complexes by catalyzing K63-linked ubiquitination of RIPK1 and themselves. LUBAC-mediated linear ubiquitination of NEMO enables TAK1-dependent IKKβ activation, leading to IκBα phosphorylation, K48-ubiquitination, degradation, and nuclear translocation of p50/RelA to drive the canonical NF-κB pathway. cIAPs depletion shifts balance to death pathways: Apoptosis: non-ubiquitinated RIPK1 forms complex IIa (RIPK1-FADD-caspase-8), activating caspase-dependent apoptosis. Necroptosis: caspase inhibition (e.g., Q-VD-Oph or Z-VAD-FMK) stabilizes RIPK1, enabling complex IIb (RIPK1–RIPK3-MLKL) for membrane permeabilization and mediating necroptosis. Non-canonical NF-κB: cIAPS loss stabilizes NIK, triggering IKKα-dependent p100 processing to p52. p52/RelB dimers activate the non-canonical NF-κB pathway

Apoptosis and necroptosis are two major forms of cell death that play central roles in oncogenesis and tumor progression. Dysregulation of these pathways enables tumor cells to evade death signals, contributing to malignant transformation, therapeutic resistance, and poor clinical outcomes. IAPs are key regulators of this balance, coordinating the decision between cell survival, apoptotic execution, and necroptotic activation. Their altered expression disrupts these regulatory networks, thereby promoting cancer development and influencing treatment response (Fig. 4) [6].

Upon TNFα binding to TNFR1, the receptor undergoes conformational rearrangement and recruits TRADD to its cytoplasmic death domain, initiating the assembly of membrane-proximal complex I. RIPK1 and TRAF2 are subsequently recruited, and cIAP1 and cIAP2 are brought to the complex through TRAF2-mediated interactions involving their BIR1 and UBA domains [53]. Within this signaling platform, cIAP1/2 function as E3 ubiquitin ligases, catalyzing auto-ubiquitination and the attachment of K11-, K63-, and K48-linked ubiquitin chains to RIPK1. In particular, K63-linked ubiquitination of RIPK1 promotes the stepwise recruitment and activation of the TAK1–TAB2/3 complex and the IKK complex via the LUBAC complex. LUBAC further generates linear ubiquitin chains on TNFR1 signaling components—including TRADD, RIPK1, and NEMO—thereby stabilizing complex I and positioning TAK1 to phosphorylate and activate IKKβ [54]. Activated IKKβ then phosphorylates IκBα on its N-terminal serine residues, targeting it for K48-linked ubiquitination and proteasomal degradation via β-TrCP/SLimb. The removal of IκBα releases the p50/RelA NF-κB heterodimer, enabling nuclear translocation and transcriptional activation of genes that regulate cell survival, inflammatory responses, and innate immunity, thereby initiating the canonical NF-κB pathway [55, 56]. In parallel, TAK1 activates MAPK signaling cascades, including JNK, p38, and ERK1/2, further broadening the cellular response to TNFR1 stimulation [57].

When cIAPs are absent, RIPK1 is no longer ubiquitinated and thus cannot be stably retained within complex I. As a result, RIPK1 dissociates into the cytosol and assembles complex II (ripoptosome) through interactions with FADD, caspase-8, and cFLIP, which acts as a structural homolog that competes with caspase-8 and limits its catalytic activity [58]. The functional outcome of the complex II platform depends on the enzymatic activity of caspase-8. When caspase-8 activity is sufficient, it cleaves and inactivates RIPK1, driving apoptosis through canonical caspase-dependent pathways. When caspase-8 is inhibited (e.g., by Q-VD-OPh or Z-VAD-FMK) or suppressed by cFLIP, RIPK1 remains uncleaved, allowing its oligomerization with RIPK3, thereby forming complex II: the ripoptosome, which initiates necroptosis [59, 60]. This necroptotic switch activates RIPK3 through autophosphorylation, enabling phosphorylation of MLKL at conserved threonine residues (T357/S358 in humans). Activated MLKL oligomerizes and translocates to cellular membranes, inducing regulated membrane permeabilization and executing necroptosis [61].

Furthermore, in the absence of cIAPs, members of the TNF receptor superfamily (TNFRSF), including BAFF-R, LTβR, CD40, TNFR2, RANK, and Fn14, activate the non-canonical NF-κB pathway [62, 63]. Under basal conditions, TRAF2 recruits cIAP1/2 to TRAF3–NIK complexes, where TRAF3 binds NIK and positions it for cIAP-mediated K48-linked ubiquitination and proteasomal degradation [64]. This process prevents NIK accumulation and maintains the non-canonical NF-κB pathway in an inactive state. When TNFRSF receptors such as CD40 or BAFF-R are stimulated, they facilitating cIAP-mediated ubiquitination and TRAF3 proteasomal degradation, which results in NIK's release from the complex. Upon cIAPs depletion, NIK is stabilized and accumulates, enabling it to phosphorylate and activate the IKKα dimer. Activated IKKα then phosphorylates p100, which causes it to be ubiquitinated and partially processed by the proteasome to make p52 [65]. The resulting p52 forms a heterodimer with RelB, and this complex translocates into the nucleus to bind κB enhancer elements and activate transcription of non-canonical NF-κB target genes [66].

### Immune modulation

During evolution, humans have developed dual immune defense systems—the innate and adaptive immunity—that operate in a coordinated manner to recognize and eliminate invading pathogens [67]. The innate immune system, serving as the host’s first line of defense, employs pattern recognition receptors (PRRs) to sense pathogen-associated molecular patterns (PAMPs) and endogenous damage-associated molecular patterns (DAMPs) [68]. These receptors are expressed by both immune and non-immune cells—including epithelial and endothelial cells, fibroblasts, dendritic cells, neutrophils, and macrophages—thereby enabling a rapid systemic response [69]. In contrast, the adaptive immune system mediates antigen-specific recognition through somatic gene rearrangement, clonal expansion, and long-lived immunological memory in T and B lymphocytes that provide precision and durability in pathogen and tumor surveillance [70].

**Fig. 5:**
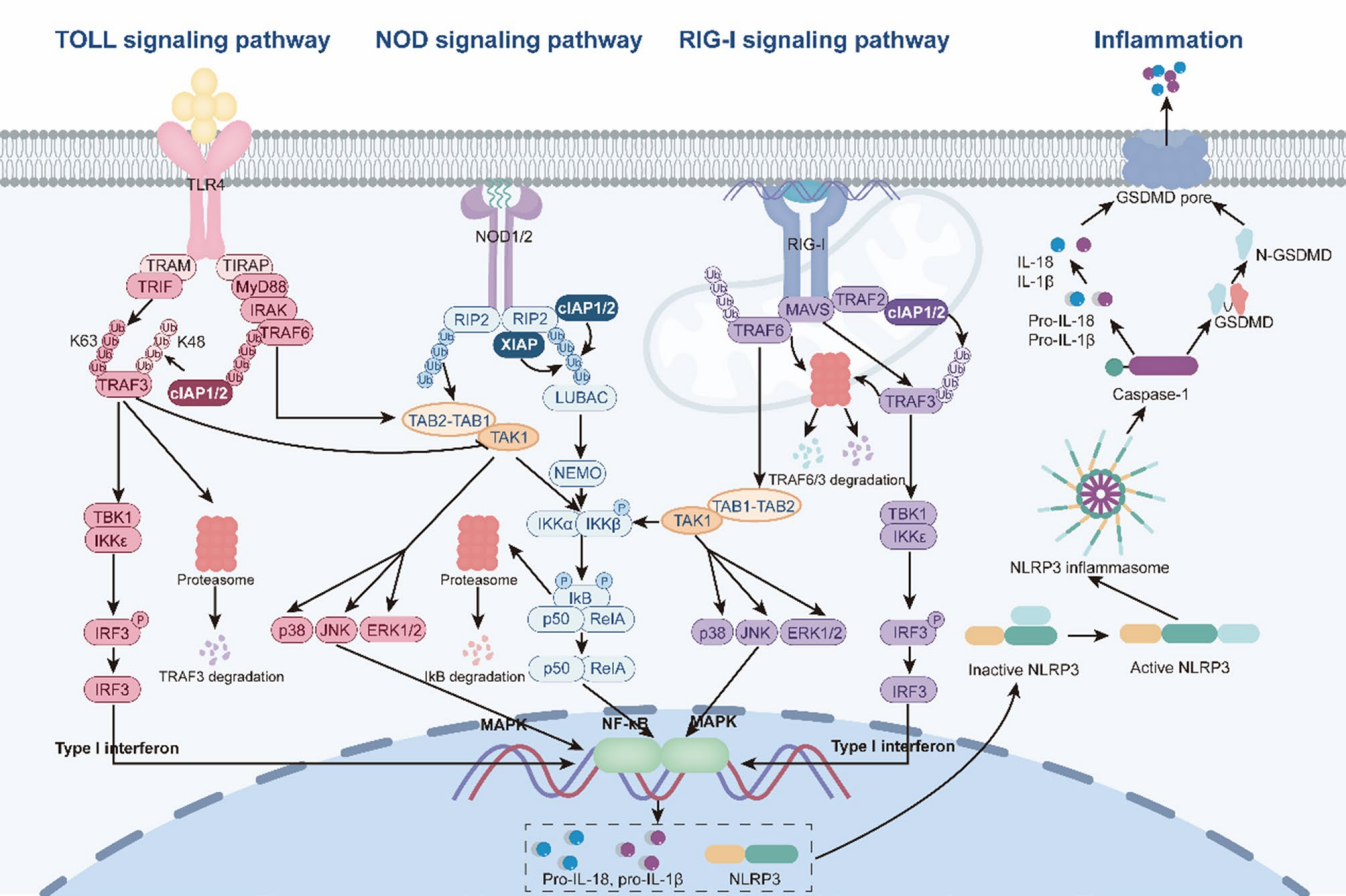
IAPs modulate the innate immune system through TLRs, NLRs, RIG-I, and the inflammasome signaling pathway. Toll signaling pathway: TLR4 binds to LPS and recruits adaptors TIRAP/TRAM to form complexes with MyD88 and TRIF. MyD88 recruits IRAK kinases, activating TRAF6, which synthesizes K63-ubiquitin chains to assemble TAB2/3-TAK1, phosphorylating IKKβ and activating NF-κB/MAPK pathways. TRIF recruits TRAF3 to generate K63-ubiquitin chains, activating TBK1/IKKε. When these kinases phosphorylate IRF3, it dimerizes, undergoes nuclear translocation, and produces IFN-β. cIAPs in the MyD88 complex mediate K48-ubiquitination and degradation of TRAF3, releasing the complex to sustain TRAF6/TAK1-driven inflammatory responses. NOD signaling pathway: Upon binding muropeptides, NOD1 and NOD2 undergo oligomerization and recruit RIP2 kinase, cIAP1/2 and XIAP. cIAP1/2 catalyze both K48- (degradation) and K63-ubiquitination (scaffolding). XIAP mediates K63-ubiquitination of RIP2 at lysines 209, 410, and 538 to stabilize RIP2. K63-chains recruit TAB1–TAB2–TAK1, which phosphorylates IKKβ (in the IKKα/β/NEMO complex) and activates MAPKs (JNK/p38/ERK1/2). XIAP and cIAP1/2 as NOD1/2 signaling positive regulators. RIG-I signaling pathway: RIG-I and MDA5 detect viral RNA, and RIG-I’s K63-linked ubiquitination by TRIM25/RNF135 enhances its interaction with MAVS. MAVS recruits TRAF3 to activate TBK1/IKKε, phosphorylating IRF3 to induce IFN production. Concurrently, TRAF6 synthesizes K63-chains, recruiting TAB2/3-TAK1 to activate IKKβ, thereby activating the NF-κB signaling. cIAP1/2 positively regulates RLR signaling. Inflammasomes: NLRs and AIM2 detect PAMPs/DAMPs via upstream receptors (TLRs/NOD/RLRs). Oligomerization recruits ASC via PYD-PYD interactions, assembling pro-caspase-1 through CARD-CARD binding. Autocatalytic cleavage activates caspase-1, which (I) processes pro-IL-1β/pro-IL-18 into bioactive forms and (II) cleaves GSDMD, releasing GSDMD-N, which oligomerizes in membranes, causing pyroptosis and cytokine release

The innate immune response is mediated through several major PRR families, including Toll-like receptors (TLRs), NOD-like receptors (NLRs), RIG-I-like receptors (RLRs), and inflammasome-forming receptors, which collectively initiate NF-κB, MAPK, and interferon (IFN) signaling cascades to drive inflammation and host protection (Fig. 5) [71].

TLRs are transmembrane pattern-recognition receptors localized on cell membranes, endosomes, and lysosomes that recognize conserved PAMPs derived from bacteria, fungi, viruses, and parasites [72] Based on ligand type and localization, surface TLRs (TLR1, −2, −4, −5, −6, −11) primarily detect microbial membrane components, whereas endosomal TLRs (TLR3, −7, −8, −9) sense nucleic acids from pathogens or host cells [72]. Upon ligand binding, TLRs initiate intracellular signaling via adapter proteins MyD88 or TRIF—with TLR4 uniquely utilizing both pathways [73]. Structurally, TLR4 contains an extracellular leucine-rich repeat (LRR) domain, a hydrophobic transmembrane helix, and an intracellular Toll/IL-1 receptor (TIR) domain [74]. Upon lipopolysaccharide (LPS) binding, TLR4 interacts with its co-receptor, MD-2, enabling TIR-TIR interactions to recruit TIRAP and TRAM, assembling a signaling platform for MyD88- and TRIF-dependent cascades [75]. In the MyD88-dependent pathway, MyD88 recruits IRAK kinases to activate TRAF6, promoting K63-linked ubiquitination that recruits the TAB2/TAB3–TAK1 complex. Activated TAK1 phosphorylates IKKβ, triggering NF-κB/MAPK activation and the transcription of inflammatory cytokines. In the TRIF-dependent pathway, TRAF3 undergoes K63-linked ubiquitination leading to activation of TBK1/IKKε, phosphorylation of IRF3, and induction of type I interferon (IFN-β) expression [76]. Notably, cIAP1/2, recruited to the MyD88 complex, mediate K48-linked ubiquitination and degradation of TRAF3. This process enables MyD88 complex dissociation and cytoplasmic translocation to sustain TRAF6–TAK1 activation and prolongs NF-κB/MAPK-mediated transcription of inflammatory cytokines, thereby fine-tuning TLR4-driven innate immune responses [77].

NLRs, including NOD1 and NOD2, are cytoplasmic PRRs characterized by an N-terminal CARD domain, a central NOD domain, and a C-terminal LRR domain, which together mediate microbial recognition and signaling [78]. They detect bacterial peptidoglycan-derived muropeptides, triggering receptor oligomerization and formation of a signaling complex with RIP2 kinase, cIAP1/2, and XIAP [79]. Within this complex, cIAP1/2 perform dual ubiquitin-editing functions, generating K63-linked scaffolds that activate signaling and K48-linked chains that mark proteins for degradation. XIAP specifically ubiquitinates RIP2 at lysine residues 209, 410, and 538, stabilizing the kinase and promoting pathway activation [80]. These polyubiquitin chains recruit the TAK1–TAB2/3 kinase complex, which phosphorylates IKKβ within the canonical IκB kinase complex (consisting of NEMO, IKKα, and IKKβ) and activates MAPK (JNK, p38, and ERK1/2) [81]. The cascades drive NF-κB translocation and MAPK-dependent transcription of chemokines and pro-inflammatory cytokines [82]. Moreover, XIAP is essential for LUBAC recruitment and linear ubiquitination of NEMO, reinforcing NF-κB activation [83]. Together, XIAP and cIAP1/2 act as positive regulators of NOD1/2 signaling [79].

RLRs, including RIG-I and MDA5, serve as cytosolic sensors of viral RNA. Upon binding single/double-stranded RNA, they activate the mitochondrial adaptor MAVS, which recruits TRAF2, TRAF3, and TRAF6 to form filamentous signaling complexes [84]. RIG-I undergoes K63-linked ubiquitination by TRIM25/RNF135, facilitating MAVS interaction and downstream recruitment of TRAF3 and TRAF6. This leads to activation of TBK1/IKKε–IRF3, inducing type I interferon (IFN-β), and TAK1–TAB2/3–IKKβ, triggering NF-κB dependent inflammatory gene expression [85]. cIAP1/2 enhance this antiviral signaling, whereas the role of XIAP remains context-dependent, with studies suggesting it may either limit virus-induced apoptosis or modulate death receptor-mediated cell death without affecting IFN production [86–91].

Inflammasomes are cytosolic multiprotein complexes assembled by specific NLR family members (NLRP1, NLRP3, NLRP6, NLRP7, NLRP12, NLRC4) and the DNA sensor AIM2, functioning as caspase-1-activating platforms that process IL-1β and IL-18 and initiate pyroptosis through Gasdermin D (GSDMD)-mediated membrane pore formation [92]. Activation begins with transcriptional upregulation of pro-IL-1β and pro-IL-18 via upstream PRRs (TLRs, NOD, RLRs, TNFRs, or IL-1 R). Subsequent receptor oligomerization recruits the adaptor ASC, which promotes CARD–CARD-mediated assembly of pro-caspase-1, leading to its autocleavage and activation [93]. Active caspase-1 cleaves both cytokine precursors and GSDMD, releasing N-GSDMD fragments that form pores and mediate inflammatory cell death, thereby driving osmotic lysis, cellular rupture, and release of mature cytokines, including mature IL-1β and IL-18 [94]. The regulatory role of IAPs in inflammasomes remains context-dependent [95]. Experimental studies have shown that cIAP1/2 can either enhance or suppress IL-1β maturation and caspase-1 activation, depending on cellular and environmental cues [96, 97]. Furthermore, treatment with SMAC mimetics in macrophages promotes NLRP3-caspase-1 and caspase-8-dependent IL-1β processing, highlighting context-dependent complex crosstalk [98].

Beyond their well-established roles in innate immunity, IAPs also regulate adaptive immune responses through multiple molecular mechanisms [99]. By reducing cIAP expression or mimicking BAFF/CD40L signaling, mediate TRAF3 degradation and subsequent NIK stabilization, thereby activating the non-canonical NF-κB pathway [100]. This axis negatively regulates B-cell proliferation and survival, linking cIAP-dependent ubiquitin editing to immune homeostasis. Additionally, cIAPs function as key negative regulators of T cell activation. Pharmacologic inhibition with SMAC mimetics enhances CD4^+^ T-cell proliferation and cytokine production via NIK accumulation and non-canonical NF-κB activation [101]. In murine models, this effect extends to CD8^+^ T cells, NKT cells, and NK cells, augmenting antigen-specific and antitumor immune responses [102]. cIAPs also exert dual functions in myeloid cells. While SMAC treatment paradoxically increases monocyte apoptosis, it promotes dendritic cell maturation via non-canonical NF-κB signaling and M2-to-M1 macrophage polarization by metabolic reprogramming [103]. This dual-effect polarization enhances TNF-α, IFN-γ, and IL-12 production and recruits neutrophils, establishing a pro-inflammatory, tumor-suppressive microenvironment [104].

Loss-of-function mutations in XIAP cause X-linked lymphoproliferative syndrome type 2 (XLP2), characterized by immune dysregulation with preserved basal immunity but heightened susceptibility to apoptosis and inflammatory disease [105]. XIAP-deficient T cells exhibit activation-induced apoptosis via FAS/CD95, TRAIL-R, or TCR signaling [106], while monocytes show impaired cytokine production (e.g., TNFα and IL-8) [105]. Reduced circulating iNKT and MAIT cells in XLP2 patients have been linked to Epstein-Barr virus (EBV) infection or chronic inflammation such as inflammatory bowel disease (IBD) and hemophagocytic lymphohistiocytosis (HLH) [107], though somes tudies report normal iNKT cell counts in both XLP2 patients and XIAP-deficient murine models [108].

Survivin is essential for T-cell proliferation, differentiation, and homeostasis via Notch-dependent signaling [109]. Conditional knockout models reveal that early Survivin deletion disrupts thymic T-cell development, whereas post-thymic deletion causes peripheral CD4^+^/CD8^+^ lymphopenia despite normal thymopoiesis [110]. Conversely, Survivin overexpression enhances effector differentiation but facilitates tumor immune evasion by modulating CD8^+^ T-cell cytotoxicity, highlighting its dual role in immune regulation and oncogenesis [111, 112].

In summary, PRRs orchestrate inflammatory cascades through NF-κB and MAPK pathways, inducing chemokines (like IL-8 and MCP1), cytokines (like TNFα, IL-1/2/6, and GM-CSF), adhesion molecules (like ICAM-1, VCAM-1, and E-selectin), acute-phase proteins/antimicrobial peptides (like SAA, and β-defensins), biosynthetic enzymes, and anti-apoptotic factors, while inflammasome-mediated pyroptosis as a conserved defense mechanism [113, 114]. Complementing this innate immune regulation, IAPs orchestrate adaptive immune balance by controlling lymphocyte proliferation, differentiation, maturation, and polarization, thus bridging cell survival, inflammation, and immune homeostasis [115].

## Clinical relevance of IAPs in cancers

IAPs are frequently overexpressed in a wide range of hematological malignancies and solid tumors, where their expression levels correlate strongly with therapeutic response and clinical prognosis, underscoring their potential as diagnostic and prognostic biomarkers (Table 1) [223].

**Table 1:** Expression profiles, prognostic outcome, and translational implications of IAPs across cancers

Cancer type	Key IAPs	Expression pattern/Localization	Expression level	Patient outcome	Therapeutic/Translational implication	Reference
Leukemia (AML, ALL, ATL)	cIAP1/2, XIAP, Survivin, BRUCE, Livin	cIAP2 elevated in AML; XIAP variable among subtypes; Survivin highly expressed in pediatric ALL and ATL	High (cIAP2, Survivin, Livin, BRUCE); Variable (XIAP)	Overexpression of cIAP2 associated with inferior OS; High XIAP expression associated with poorer chemotherapy responses and reduced RFS in pediatric AML; High Survivin or Survivin-2B/DEx2 isoform ratios associated with shortened OS and early relapse in AML; Elevated Livin and BRUCE expression associate with poor OS in AML; Increased Survivin mRNA associated with shorter survival and enhanced malignant potential in ATL	High multiple IAPs expression predicts lower response to daunorubicin/cytarabine (3 + 7) chemotherapy; Elevated XIAPs associated with decreased responses to prednisone in ALL	[115–126]
Colorectal cancer (CRC)	NAIP, cIAP1/2, XIAP, Survivin, BRUCE, Livin	NAIP reduced in tumor; cIAP1 predominantly nuclear; cIAP2 nuclear and cytoplasmic; Survivin mainly cytoplasmic	Low (NAIP); High (cIAP1/2, XIAP, Survivin); Variable (Livin)	Nuclear cIAP1 correlated with higher mortality; Cytoplasmic cIAP1/2 correlated with better survival; Nuclear cIAP2 correlated with stromal lymphocytic infiltration; XIAP mRNA overexpression associates with lymph node metastasis; Survivin linked to lymph-node metastasis and poorer differentiation; Increased BRUCE expression associated with adverse clinicopathologic features	Down-regulation of cIAP2 increases apoptosis and enhances 5-FU sensitivity	[115, 127–134]
Breast cancer (including TNBC)	cIAP2, XIAP, Survivin, BRUCE	XIAP and Survivin up-regulated in advanced stages	High	Nuclear XIAP/Survivin associated with shorter OS and DFS; cIAP2 overexpression is associated with lymph node-positive cases; XIAP and BRUCE overexpression associated with a lower OS in TNBC; Survivin overexpression is associated with metastasis; Cytoplasmic Survivin correlated with lymph-node metastasis; Survivin scores also correlate with elevated mortality rates	BRUCE silencing increases cisplatin and paclitaxel sensitivity	[135–145]
Hepatocellular carcinoma (HCC)	NAIP, cIAP1/2, XIAP, Survivin, Livin	XIAP reduced in poorly differentiated tumors; Survivin and Livin mRNA levels are upregulated in tumors; Survivin mRNA correlated with advanced stage	Low (XIAP) High (NAIP, Survivin, Livin)	cIAP1 mRNA overexpression is associated with patient age; XIAP overexpression associated with reduced OS; Elevated Survivin mRNA associated with vascular invasion; Cytoplasmic Survivin associated with shorter OS and RFS	Survivin serves as a strong predictor of HCC patients	[146–148]
Prostate cancer	NAIP cIAP1/2, XIAP, Survivin, BRUCE	Survivin overexpressed in tumors; BRUCE increased in advanced stages	High	BRUCE overexpression associated with poor clinical outcome and metastasis	Survivin inhibition enhances paclitaxel and docetaxel chemotherapy efficacy; BRUCE regulates doxorubicin sensitivity	[149–154]
Esophageal cancer (ESCC)	XIAP, Survivin, BRUCE	XIAP, Survivin and BRUCE highly expressed in tumors	High	High XIAP and Survivin associated with poor prognosis and chemotherapy resistance; XIAP and BRUCE correlated with advanced stage; BRUCE expression correlated with invasion depth	Lower Survivin expression observed in cisplatin/5-FU responders	[155–159]
Oral cancer (OSCC)	Survivin, Livin	Survivin and Livin upregulated in tumors; Cytoplasmic Survivin positive in > 95% of cases	High	High Survivin expression associated with short OS and advanced stage; Nuclear Survivin correlated with advanced stage and lymph-node metastasis; Cytoplasmic Survivin as an independent prognostic marker for reduced OS and chemoradiotherapy resistance; Livin as a marker of poor prognosis	Survivin knockdown enhanced radiosensitivity and increased OS; Livin silencing improved radiosensitivity and chemosensitivity, and prolong survival	[160–167]
Bladder cancer (TCC/NMIBC)	NAIP, cIAP1/2, XIAP, Survivin, BRUCE, Livin, hILP2	NAIP, Survivin, BRUCE, Livin, and hILP2 up-regulated significantly in tumors; XIAP and Survivin variable among tumors;	High (NAIP, Survivin, BRUCE, Livin, hILP2); Variable (XIAP, Survivin)	Nuclear Survivin correlated with grade, stage and prognosis, but its histological clinical association remains controversial; Livin upregulation associated with early recurrence and significantly shorter median RFS; Survivin and Livin combined predict prolonged RFS	Survivin as a potential biomarker; Urinary Survivin serves as non-invasive marker for recurrence monitoring and early detection	[168–179]
Lung cancer (NSCLC)	cIAP1/2, XIAP, Survivin, Livin	cIAP1, cIAP2, XIAP, and Survivin overexpressed in tumors; cIAP1 and Survivin detected in nucleus and cytoplasm; cIAP2 and XIAP primarily cytoplasm	High	Cytoplasmic Survivin associated with poor differentiation in NSCLC; Nuclear Survivin expression associated with advanced stage, reduced 5-year OS and as a prognostic biomarker for OS	XIAP linked to radio- and chemo-resistance; Nuclear survivin correlates with lymph node metastasis; Survivin overexpression promotes tumor angiogenesis and metastasis in SCLC; Combined inhibition of Livin and Survivin shows synergistic antitumor effects	[180–188]
Osteosarcoma	Survivin	Survivin overexpressed in tumors	High	Nuclear Survivin associated with larger tumor size and shorter survival period	Survivin silencing increases sensitivity to etoposide, cisplatin, and doxorubicin; Survivin knockdown increases T cell sensitivity in RMS	[189, 190]
Melanoma (uveal and melanoma)	Survivin, Livin	Survivin and Livin overexpressed in tumors	High	Elevated Survivin expression associated with poorer survival	Elevated Survivin correlate with adjuvant immunotherapy; Livin expression linked to Adriamycin- and 4-TBP-induced apoptosis resistance	[191–194]
Glioma (LGG/HGG)	cIAP2, Survivin	cIAP2 increased in high-grade glioma; Survivin consistently overexpressed	High	cIAP2 expression associated with short survival; Survivin promotes angiogenesis	Elevated cIAP2 and Survivin inhibits apoptosis and promotes cell proliferation	[195–198]
Ovarian cancer	XIAP, Survivin, BRUCE	XIAP and BRUCE up-regulated in ovarian cancer; Survivin progressively increases from benign to malignant lesions	High	Survivin linked to higher mortality; Nuclear Survivin identified as independent predictor of poor OS and DFS; BRUCE expression served as an independent predictor of reduced OS and DFS	XIAP associated with cisplatin-induced chemoresistance; Survivin useful for stage risk stratification	[199–203]
Renal cell carcinoma (RCC)	cIAP1/2, XIAP, Survivin variants, BRUCE	cIAP1/2 and BRUCE overexpressed in tumors; cIAP1/2 higher in early stages; XIAP expression rises with tumor stage	High (XIAP); Variable (cIAP1/2)	Reduced cIAP1 correlates with shorter RFS and survival periods; Elevated XIAP expression predicts poor postoperative survival, while low XIAP expression correlated with longer disease-specific survival; BRUCE overexpression linked to shortened OS	BRUCE overexpression linked to lymph node metastasis, advanced T/TNM stages	[204–209]
Gastric cancer	cIAP2, XIAP, Survivin, BRUCE	cIAP2 overexpressed in > 70% of tumors; XIAP and Survivin overexpressed in tumors; BRUCE displayed gender-dependent expression	High	XIAP reduction associated with tumor differentiation and lymph node infiltration; Cytoplasmic Survivin expression is enriched at the invasive tumor margins; Survivin-2B mRNA negatively correlated with tumor stage and invasion depth	cIAP2 silencing promotes apoptosis, inhibits proliferation, and reduces migratory; XIAP down-regulation enhances cisplatin sensitivity; High XIAP expression associated with lower metastatic potential	[210–216]
Pancreatic cancer	cIAP2, XIAP, Survivin (α/2B/ΔEx3)	cIAP2 and Survivin isoforms up-regulated in tumors; XIAP overexpressed in 77% of primary cancers	High	Nuclear Survivin correlated with poor OS and DFS; Nuclear Survivin expression as an independent prognostic biomarker for OS in pancreatic tail cancer and DFS in pancreatic ductal adenocarcinoma; Cytoplasmic Survivin correlates with venous infiltration; Nuclear Survivin is linked to perineural invasion	cIAP2 expression related to cisplatin and paclitaxel sensitivity; Survivin-2B overexpression linked to gemcitabine resistance; Cytoplasmic Survivin associated with metastasis and advanced stage	[217–220]
Neuroblastoma	Survivin, Livin	Survivin consistently expressed across all neuroblastoma cell lines	High	Co-expression of Livin and MYCN amplification associated with poor survival and as a combined prognostic biomarker	Survivin expression correlated with cell proliferation and cell cycle	[221, 222]

Expression level definitions: High = up-regulated relative to normal; Low = down-regulated; Variable = heterogeneous or subtype-dependent

Abbreviations: AML: acute myeloid leukemia; ALL: acute lymphoblastic leukemia; ATL: adult T-cell leukemia; cIAP: cellular IAP; XIAP: X-linked IAP; CR: complete remission; OS: overall survival; DFS: disease-free survival; RFS: recurrence-free survival; ESCC: esophageal squamous cell carcinoma; HCC: hepatocellular carcinoma; NMIBC: non-muscle-invasive bladder cancer; OSCC: oral squamous cell carcinoma; PDAC: pancreatic ductal adenocarcinoma; RCC: renal cell carcinoma; TNBC: triple-negative breast cancer

### Leukemia

Leukemia is the most common malignancy in children, accounting for 3%–4% of all new cancer cases and 3%–5% of cancer-related deaths worldwide, and is classified into myeloid and lymphoid subtypes according to hematopoietic lineage origin [224, 225].

In acute myeloid leukemia (AML), the overexpression of cIAP2 has been linked to inferior OS [116]. XIAP expression varies across AML subtypes—overexpressed in MOLT4 but reduced in HL60 cells—with conflicting evidence regarding its prognostic significance: some studies associated low XIAP expression with prolonged OS [117], whereas others found no independent predictive value for complete remission (CR) rates or OS [118]. Furthermore, differential regulation of XIAP during myelopoiesis has been demonstrated, displaying upregulation during monocytogenesis but not granulocytic differentiation [119]. Importantly, pediatric AML patients with high XIAP expression exhibit poorer early chemotherapy responses and reduced 3-year recurrence-free survival (RFS) [120]. Survivin, detected in most myeloid leukemia cell lines, consistently correlates with unfavorable outcomes. High Survivin or Survivin-2B/DEx2 isoform ratios predict shortened OS and early relapse, particularly in pediatric AML [121]. In relapsed/refractory diffuse large B-cell lymphoma (R/R DLBCL) treated with pembrolizumab, cyclophosphamide, and maveropepimut-S immunotherapy, high-intensity Survivin expression is strongly associated with aggressive clinical features [122]. Similarly, elevated Livin and BRUCE expression independently associate with poor OS in both adult and pediatric AML patients, characterized by increased relapse risks, chemotherapy resistance, and reduced survival [115]. Combinatorial analyses further demonstrate that AML patients expressing multiple IAPs (XIAP, cIAP1, cIAP2, and Survivin) show a stepwise decline in chemotherapy response to those containing daunorubicin and cytarabine (3+7 protocol), with CR rates decreasing from 100% in low-expression cases to 33% in those overexpressing all four IAPs [123].

In pediatric acute lymphocytic leukemia (ALL), high-risk and relapsed patients exhibit significantly increased Survivin expression compared to standard-risk or remission groups. Livin expression serves as an independent prognostic factor, and elevated XIAP levels correlate with reduced prednisone responsiveness [124]. In adult T-cell leukemia (ATL), Survivin is consistently overexpressed across subtypes, with the highest levels in aggressive variants and cell lines, where increased Survivin mRNA predicts shorter survival and enhanced malignant potential [125]. Furthermore, patients with disease progression also display concurrent overexpression of cIAP1/2, XIAP, and Survivin, which collectively associate with reduced spontaneous apoptosis and poor prognosis [115]. Notably, co-expression of Survivin and cIAP1 identifies a subgroup of leukemia patients with significantly shorter OS, highlighting their combined prognostic relevance [124].

### Colorectal cancer (CRC)

Colorectal cancer (CRC) is the third most commonly diagnosed cancer worldwide and the second leading cause of cancer-related mortality, accounting for approximately 10% of new cancer cases and 9%-10% of cancer deaths globally [225].

In CRC, multiple members of the IAP family demonstrate aberrant expression patterns that correlate with patient prognosis and treatment response. NAIP expression is significantly reduced in tumor tissues compared with normal colonic mucosa, with lower levels in older patients, suggesting that there exists an age-related decline [127]. Both cIAP1 and cIAP2 are broadly expressed in CRC cell lines and tissue specimens, displaying distinct subcellular localization: cIAP1 is predominantly nuclear, and cIAP2 is distributed between the cytoplasm and nucleus [115, 128]. Clinically, cytoplasmic cIAP1/2 expression is associated with improved survival, whereas nuclear cIAP1 correlates with higher mortality. Nuclear cIAP2 expression correlated with intense stromal lymphocytic infiltration, and cIAP2 downregulation enhances apoptosis and increases chemosensitivity to 5-fluorouracil (5-FU) [129]. XIAP mRNA levels are frequently elevated in CRC tissues relative to normal mucosa and adenomas, and its overexpression associates with tumor stage, lymph node metastasis, and increased aggressiveness, though some studies report variable expression trends [130]. Survivin is consistently overexpressed in colorectal adenocarcinomas and CRC cell lines compared with adjacent non-tumor tissues, showing strong cytoplasmic localization correlated with lymph node metastasis, advanced age, and poorer differentiation [131, 132]. Evidence regarding Livin remains inconsistent: while some studies suggest its expression predicts favorable outcomes [133], others report no significant overexpression in malignant tissues but rather increased BRUCE expression in non-tumor samples associated with adverse clinicopathologic features [130, 134].

### Breast cancer

Breast cancer is the most common malignancy worldwide, accounting for 24–25% of female-specific cancers and 6.9% of global cancer-related mortality, making it the second leading cause of death among women [225].

In breast cancer, advanced-stage tumors had significantly higher levels of XIAP and Survivin expression, with XIAP detected in 99% of patients and exhibiting markedly higher median expression compared to normal controls [135]. Low XIAP expression correlated with prolonged progression-free survival (PFS), whereas nuclear XIAP protein localization was independently associated with shorter OS [136, 137]. Concurrently, cIAP2 overexpression was predominantly observed in lymph node-positive cases [115]. In patients with triple-negative breast cancer (TNBC), elevated levels of the XIAP and BRUCE genes were independently associated with a lower OS, particularly in those lacking lymphovascular or fat tissue invasion, where NAIP, cIAP1, cIAP2, Survivin, BRUCE, and hILP2 are markedly upregulated [138]. Survivin expression is consistently higher in malignant tissues than in normal counterparts, showing strong associations with disease progression, metastasis, and adverse clinicopathological features [139]. Nuclear Survivin localization functions as an independent prognostic factor for both DFS and OS [140], whereas cytoplasmic Survivin was associated with lymph node metastasis, histological grade, and tumor stage [141]. Notably, high Survivin scores also correlate with elevated mortality rates, with 46.5% (79/169) of deceased patients expressing Survivin compared to only 19.4% (33/170) among Survivin-negative cases, while among survivors, Survivin positivity was observed in only 5.9% (10 cases) compared with 28.2% (48 cases) in Survivin-negative counterparts [142], Moreover, Survivin overexpression synergizes with Ki67 to promote tumor cell proliferation [143]. Functionally, BRUCE knockdown enhanced chemosensitivity to cisplatin or paclitaxel in breast cancer models [144], while elevated Livin expression is associated with highly aggressive tumor phenotypes [145].

### Hepatocellular cancer (HCC)

Hepatocellular carcinoma (HCC) accounts for 4.7% of all new cancer diagnoses and 8.3% of cancer-related deaths globally, ranking as the second leading cause of cancer mortality with a notably higher mortality in males [225].

In HCC, dysregulated expressions of multiple IAPs correlate strongly with tumor progression and patient outcomes. Elevated NAIP mRNA levels exhibit a significant association with pseudoglandular histological subtypes, while cIAP1 mRNA overexpression is associated with younger patient age, and cIAP2 mRNA upregulation correlates with tumors lacking a fibrous capsule. Increased XIAP expression is consistently related to shortened OS [146]. Conversely, XIAP expression is significantly reduced in poorly differentiated tumors, suggesting a context-dependent regulatory role during tumor differentiation [147]. Both Survivin and Livin mRNA levels are markedly upregulated in malignant tissues compared to adjacent non-tumor, with Survivin mRNA expression positively correlating with advanced stage, higher histological grade, and vascular invasion [146]. Importantly, cytoplasmic localization of Survivin in tumor cells serves as a strong predictor of poor outcomes, correlating with reduced OS and RFS rates in HCC patients [148].

### Prostate cancer

Prostate cancer is the second most common cancer in males, accounting for 15% of all male cancers and causing an estimated 375,000 deaths globally in 2020 [225].

Multiple members of the IAP family—including NAIP, cIAP1, cIAP2, XIAP, and Survivin—are consistently expressed at both mRNA and protein levels across common prostate cancer cell lines (LNCaP, PC3, DU145) [149–151]. Among these, Survivin is scarcely detectable in normal prostate tissues but markedly overexpressed in malignant lesions [152]. Its expression is further enhanced following docetaxel treatment, while inhibition of Survivin synergistically enhances the cytotoxic effects of paclitaxel and docetaxel by promoting caspase activation, apoptosis, and reversal of chemoresistance [153]. Similarly, BRUCE expression is markedly increased in prostate tumor tissues relative to benign counterparts, with even higher expression observed in advanced-stage disease [151]. Elevated BRUCE serves as an independent prognostic biomarker associated with poor clinical outcomes, metastatic progression, and higher pathological stage. Functional studies support its role in regulating cell proliferation, apoptosis, and response to doxorubicin sensitivity [151, 154].

### Esophageal cancer

Esophageal cancer accounts for approximately 3%-4% of global cancer cases and 5–6% of cancer-related deaths, with a markedly higher incidence in males, largely attributable to lifestyle-associated risk factors such as tobacco and alcohol use [225].

In esophageal squamous cell carcinoma (ESCC), aberrant expression of several IAPs is closely associated with tumor progression and clinical outcome. XIAP expression is markedly increased in tumor tissues compared with normal tissues and correlates with advanced tumor stage, female sex, and poor prognosis [155, 156]. High XIAP levels inhibit apoptosis and reduce chemosensitivity to agents including paclitaxel, cisplatin, fluorouracil, and etoposide, contributing to treatment resistance and reduced survival [155, 156].

Survivin is significantly upregulated in ESCC, displaying a stepwise increase from benign lesions to poorly differentiated carcinomas [157]. Its overexpression inversely correlates with apoptotic cell proportions in tumor microenvironments [157] and is associated with drug resistance, early recurrence, and shortened median survival—declining from 30.0 months in low-Survivin patients to 9.0 months in high-Survivin cases [158]. In clinical cohorts receiving cisplatin/5-fluorouracil therapy, Survivin levels were significantly lower in partial remission (PR) responders than in non-responders (NC/PD) [158].

BRUCE expression is upregulated in ESCC tissues relative to normal esophageal epithelium and correlates positively with tumor invasion depth (T3/T4 vs. T1/T2) and advanced pathological stage (III/IV vs. I/II) [159].

### Oral cancer

Head and neck cancer ranks as the sixth most common malignant tumor worldwide, accounting for approximately 2–4% of all cancers, with squamous cell carcinoma (SCC) comprising about 90% of oral malignancies. Oral squamous cell carcinoma (OSCC) accounts for 2–3% of new cancer cases annually, with the highest burden observed in South and Southeast Asia and Eastern Europe [225].

In OSCC, Survivin expression is markedly elevated compared with normal oral mucosa, showing cytoplasmic positivity in nearly all dysplasia and carcinoma tissues (≈97–98%), whereas normal mucosa lacks detectable expression [160]. High Survivin levels strongly correlate with shortened OS, and Kaplan–Meier analyses confirm significantly poorer prognosis in patients with high Survivin labeling indices ( > 25%) [160]. Clinically, nuclear Survivin localization is associated with advanced tumor stage and lymph node metastasis, while cytoplasmic Survivin acts as an independent prognostic marker for reduced OS and chemoradiotherapy resistance [161, 162]. Functional studies corroborate these findings, showing that Survivin knockdown enhances apoptosis, inhibits proliferation, and increases radiosensitivity [163]. Similarly, the Livin expression is significantly upregulated in OSCC tissues relative to normal mucosa [164]. High levels of Livin correlate with tumor aggressiveness, radio- and chemoresistance, and shortened survival, identifying it as a marker of poor prognosis [165, 166].

### Bladder cancer

Bladder cancer ranks as the seventh most common malignancy in men, accounting for 3%-4% of new cancer diagnoses globally, with particularly high incidence and recurrence rates in China, where it is the most prevalent urogenital tumor in males [225].

While cIAP1, cIAP2, and XIAP exhibit baseline expression in normal bladder urothelium [168], malignant tissues show broad upregulation of NAIP, Survivin, BRUCE, Livin, and hILP2 [169]. Although most studies report reduced XIAP expression in bladder tumors, others describe strong XIAP positivity in up to 73% of urothelial carcinomas, reflecting tumor heterogeneity [170]. Collectively, elevated IAPs expression correlates with high-grade tumors and poor outcomes, emphasizing their contribution to cell survival and apoptotic resistance [171].

Among these, Survivin expression varies significantly in bladder transitional cell carcinoma (TCC). Its nuclear localization, rather than cytoplasmic distribution, correlates directly with tumor grade, stage, and prognosis [172]. Nuclear cIAP1 and Survivin levels further correlate with tumor progression [168]. However, the relationship between Survivin expression and histological clinical stage remains controversial: one study reported a 78% positivity rate (28/36) in TCC cases, with expression increasing alongside histological grades [173, 174], another found no significant correlation between Survivin levels and pathological grade or stage, while other analyses confirmed Survivin overexpression in TCC tissues associated with histological grade but not clinical stage [174, 175]. Functionally, urinary Survivin serves as a non-invasive biomarker for early detection and recurrence monitoring, with it positively correlating with tumor stage, lymph node status, and cytology, offering superior sensitivity to cytology for low-grade/early-stage patients and comparable sensitivity for high-grade/advanced cases [176]. In conclusion, Survivin is a potential biomarker in the diagnosis of bladder cancer [177].

Livin is minimally expressed in normal urothelium but markedly upregulated in non-muscle-invasive bladder cancer (NMIBC), with expression in 75.0% of cases [178]. High Livin expression is significantly associated with early recurrence and shorter median RFS, while low Survivin and Livin levels independently predict prolonged RFS, confirming their combined prognostic value [179].

### Lung cancer

Lung cancer is the second most commonly diagnosed malignancy worldwide, accounting for 11.4% of new cancer cases and remaining the leading cause of cancer-related mortality, accounting for 18.7% of global cancer deaths, particularly among males [225].

In lung cancer, multiple IAPs, including cIAP1, cIAP2, XIAP, and Survivin, are markedly overexpressed in tumor tissues compared to adjacent normal lung epithelia [180]. cIAP1 and Survivin are detected in both nuclear and cytoplasmic localization, whereas cIAP2 and XIAP expression are primarily cytoplasmic [181]. Elevated XIAP levels correlate with resistance to chemotherapy and radiotherapy, underscoring its contribution to treatment failure and poor outcomes [182]. Survivin expression exhibits distinct prognostic relevance depending on its subcellular localization. Cytoplasmic Survivin is associated with poor differentiation in non-small cell lung cancer (NSCLC) [183]. While nuclear Survivin expression correlates with advanced clinical stage, lymph node metastasis, and 5-year survival rates [182], identifying it as an independent negative prognostic biomarker for OS [184, 185]. Survivin mRNA levels are significantly higher in lung squamous cell carcinoma (LSCC) compared to lung adenocarcinoma (LAC), particularly in poorly differentiated tumors [186]. In small-cell lung cancer (SCLC), Survivin overexpression further promotes tumor angiogenesis and metastasis, reinforcing its role in disease aggressiveness [187]. Additionally, Livin is co-expressed with Survivin and XIAP in lung cancer tissues, where functional studies demonstrate that these IAPs cooperatively suppress apoptosis and enhance tumor growth [186, 188]. Combined targeting of Livin and Survivin produces a synergistic antitumor effect, suggesting translational potential for dual-IAP inhibition strategies in lung cancer therapy [188].

### Osteosarcoma

Osteosarcoma is the most common primary malignant bone tumor in children and adolescents, accounting for 0.2%–0.3% of all cancers and 0.1%–0.2% of cancer-related deaths worldwide [225].

Among IAPs, Survivin plays a central role in osteosarcoma biology and prognosis. Elevated Survivin expression is observed in osteosarcoma tissues and cell lines, where its nuclear expression correlates strongly with larger tumor size and shortened patient survival [189]. Functionally, its upregulation enhances tumor cell resistance to apoptosis, while Survivin inhibition restores chemosensitivity to agents such as etoposide, cisplatin, and doxorubicin, highlighting its translational potential as a therapeutic target [190] Downregulation of Survivin in rhabdomyosarcoma (RMS) cells increases its sensitivity to T cell [226].

### Melanoma

Melanoma accounts for 1%-2% of global cancer diagnoses and 0.6%-0.7% of cancer-related deaths, representing the most lethal form of skin malignancies, with Australia exhibiting the highest incidence worldwide [225].

Among the IAPs, Survivin and Livin are universally expressed in melanoma cell lines, with elevated Survivin levels in recurrent and metastatic melanoma correlating with poorer survival and reduced response to postoperative adjuvant immunotherapy [191]. Livin demonstrates differential expression across melanocytic lesions, with low positivity in benign nevi (10.0–21.4%) and markedly higher expression in malignant melanomas (48–71%) [192]. The differential expression of Livin between primary and metastatic melanomas remains controversial. Some studies report no significant difference in Livin expression between primary and metastatic melanomas [192]. However, in uveal melanoma, both Survivin and Livin are markedly upregulated, with Livin mRNA showing particularly higher levels in metastatic lesions. These findings suggest a potential role for Livin in tumor progression and metastatic potential [193]. Furthermore, Livin-positive tumors display greater resistance to Adriamycin- and 4-TBP-induced apoptosis, supporting its role in therapy resistance and tumor aggressiveness [194].

### Glioma

Gliomas represent the most common primary brain tumors, with glioblastoma constituting the most aggressive subtype, characterized by a five-year survival rate of only 5%-10% and a median survival time of 12–15 months [225].

Among the IAPs, cIAP2 expression increases progressively from low-grade (LGG) to high-grade gliomas (HGG), correlating with reduced patient survival and enhanced tumor cell proliferation through its anti-apoptotic activity [195]. Similarly, Survivin is markedly overexpressed in gliomas [196]. Elevated Survivin expression promotes tumor progression and adaptive evolution by supporting cell proliferation, angiogenesis, and apoptosis inhibition [197, 198].

### Ovarian cancer

Ovarian cancer accounts for 3–4% of all female cancer diagnoses worldwide but remains the leading cause of gynecologic cancer mortality, largely due to its asymptomatic onset and late-stage diagnosis, with over 70% of patients presenting at stage III or IV and a mortality rate exceeding 60% [225, 227].

XIAP is markedly upregulated in ovarian cancer tissues compared to normal ovarian tissues [199]. Experimental evidence indicates that XIAP plays a pivotal role in determining cisplatin-induced chemoresistance. In cisplatin-sensitive ovarian cancer cells, treatment induces apoptosis through downregulation of XIAP and BIRC2 protein levels, whereas resistant cells fail to initiate apoptosis and maintain high XIAP/BIRC2 expression. Moreover, XIAP overexpression attenuates cisplatin-induced reductions in XIAP protein levels and suppresses apoptotic nuclear fragmentation, underscoring its role in chemoresistance [200]. Survivin is absent in normal ovarian surface epithelium but progressively increases from benign (21.2%, 7/33) to borderline (47.8%, 11/23) and malignant tumors (51.1%, 24/47), correlating with higher proliferation indices, larger residual tumor size, and approximately 60% higher mortality risk [201, 202]. Moreover, nuclear Survivin expression emerges as an independent prognostic factor for poor OS and DFS, showing stage-dependent accumulation (stage III > I/II) and enrichment in deceased patients [203]. Similarly, BRUCE protein is significantly elevated in ovarian cancer, particularly in poorly differentiated tumors, and independently predicts reduced OS and DFS [203].

### Renal cell cancer (RCC)

RCC accounts for approximately 2%-3% of all cancer diagnoses worldwide and contributes to nearly 1.8% of all cancer-related deaths, with an estimated 400,000 new cases annually [225].

Dysregulation of IAP members is closely associated with RCC progression and patient prognosis. Both cIAP1 and cIAP2 are markedly overexpressed in RCC tissues compared to normal renal tissues [204]. Notably, higher cIAP1/2 expression is observed in stage I tumors compared with pT2/pT3 lesions, and reduced cIAP1 expression correlates with shorter RFS and cancer-specific survival periods [205]. XIAP, detected in approximately 95% of clear cell RCCs, shows progressive upregulation from well-differentiated (G1) to poorly differentiated (G3) tumors and from early (pT1) to advanced (pT3) stages [206], with stage III/IV RCCs showing 2.5-fold increased XIAP levels compared to stage I/II counterparts [207]. Elevated XIAP expression independently predicts poor postoperative survival, while lower levels are associated with prolonged postoperative disease-specific survival [206]. Survivin variants (ΔEx3, 2B, and wild type) are consistently expressed across RCC subtypes, showing no significant variant expression differences between clear cell and chromophobe/papillary RCCs [208]. BRUCE is also upregulated in RCC, and its overexpression significantly associates with lymph node metastasis, advanced TNM stages, and reduced OS [210].

### Gastric cancer

Gastric cancer accounts for 5.6% of global cancer diagnoses and ranks third in cancer-related mortality, responsible for 7.7% of all cancer deaths worldwide [225].

Among the IAPs, cIAP2, XIAP, Survivin, and BRUCE are markedly upregulated in gastric cancer tissues and cell lines compared with normal gastric mucosa. cIAP2 overexpression, observed in more than 70% of human gastric cancers, correlates with tumor differentiation status, while its silencing promotes apoptosis, inhibits proliferation, and reduces migratory capacity in gastric cancer models [211]. XIAP and Survivin show significantly elevated expression in gastric adenocarcinoma and cell lines relative to non-tumor controls [212], XIAP knockdown enhances cisplatin sensitivity and induces apoptosis in p53-wild-type cells, whereas clinically, high XIAP expression is associated with well-differentiated tumors and lower metastatic potential [213, 214]. Cytoplasmic Survivin expression is enriched at the invasive tumor margins, particularly in patients without lymph node metastasis [215]. Furthermore, Survivin splice variants display distinct prognostic relevance: Survivin 2B mRNA is inversely correlated with tumor stage and invasion depth, whereas Survivin-ΔEx3 mRNA is negatively associated with apoptosis [216]. BRUCE is detected in 98.07% of gastric cancer tissues, with a higher prevalence in female patients, although its expression shows no significant correlation with other clinicopathological variables [217].

### Pancreatic cancer

Pancreatic cancer accounts for 2%-3% of all global cancer diagnoses, representing nearly 500,000 new cases annually, and remains one of the deadliest malignancies, with the lowest five-year survival rate of only 10%-13% and a mortality-to-incidence ratio approaching 98% [225].

Among inhibitor of apoptosis proteins (IAPs), cIAP2, Survivin, and XIAP are markedly upregulated in pancreatic cancer. cIAP2 mRNA is consistently elevated in almost all pancreatic cancer cells and tumor specimens compared to normal epithelium, with expression levels significantly correlating with chemotherapeutic sensitivities, particularly to cisplatin and paclitaxel [218]. Survivin isoforms (Survivin-α, Survivin-2B, and Survivin-ΔEx3) show pronounced upregulation in pancreatic cancer cell lines, with Survivin-α and Survivin-ΔEx3 being the most dominant variants [218]. Clinically, Survivin-2B overexpression is associated with gemcitabine resistance, while cytoplasmic Survivin enrichment in tumor cores correlates with distant metastasis and advanced UICC III/IV stages [219, 220]. In contrast, high nuclear Survivin expression serves as an independent prognostic biomarker for OS in pancreatic tail cancer and DFS in pancreatic ductal adenocarcinoma (PDAC) [220]. At invasive fronts, cytoplasmic Survivin correlates with venous infiltration, whereas nuclear Survivin is linked to perineural invasion [220]. Furthermore, XIAP—rarely expressed in non-tumorous pancreatic ducts—is aberrantly upregulated in approximately 77% of primary pancreatic cancers, highlighting its tumor-specific activation [221].

### Neuroblastoma

Neuroblastoma accounts for 6%-10% of all pediatric cancers, with an annual incidence of approximately 10 cases per million children, represents the most common extracranial solid tumor in childhood, and accounts for nearly 15% of pediatric cancer-related deaths [225].

Among IAPs, Survivin and Livin demonstrate key prognostic significance in neuroblastoma. Survivin is consistently expressed across all neuroblastoma cell lines, with expression levels correlating positively with cell proliferation and cell cycle progression. High Survivin expression is associated with increased mitotic activity (G2/M phase accumulation) and enhanced tumor growth, whereas low Survivin expression corresponds to cell cycle arrest (G0/G1 phase) and reduced proliferation, underscoring its link to tumor aggressiveness [222]. While Livin expression alone does not independently predict patient survival, its co-expression with MYCN amplification significantly worsens survival outcomes. This synergistic interaction identifies a high-risk subgroup characterized by poor prognosis, suggesting that concurrent Livin upregulation and MYCN amplification may serve as a combined prognostic biomarker in neuroblastoma [228].

## Drug development and translational applications

Considering that IAPs are highly expressed in tumors and involved in multiple biological processes, which correlates with a poor prognosis for tumors, IAP inhibitors have been extensively studied (Table 2) [223]. In addition, to further emphasize its clinical application, we have summarized its clinical trials (Table 3).

**Table 2:** IAP-targeted therapeutic strategies: target, mechanisms, preclinical findings, and clinical translation

Category	Compound/Strategy	Primary IAP Targets	Mechanism of Action	Key Preclinical Findings	Clinical Stage/Trials ID	Clinical Outcomes	References
SMAC mimetic	GDC-0152 (RG7419)	cIAP1/2, XIAP, Livin	Induces cIAP degradation; activates caspase-3/7 and inhibits PI3K/AKT pathway	Inhibits growth and promotes apoptosis in melanoma, glioblastoma (hypoxic stem like cells), and breast cancer xenografts; synergy with TNF-α released from tumor-associated immune cells	Phase I (NCT00977067)	limited objective responses	[229–238].
	LCL161	cIAP1/2, XIAP	Disrupts IAP–caspase interaction; promotes IAP degradation; activates caspase dependent apoptosis; modulates NF-κB and PI3K/AKT signaling; triggers apoptosis/necroptosis; regulates ABCB1 and Survivin	Broad pro-apoptotic and antiproliferative activity; enhances paclitaxel-induced apoptosis in NSCLC; synergizes with FasL and sensitizes to radiotherapy (HNSCC); co-targeting Bcl-2 (SC-2001) overcomes resistance (HCC); radiosensitizes ESCC; synergistic with VSVΔ51-GFP and AAVP-TNF-α; potentiates efficacy with KIT, proteasome, and chemo agents (GI cancers); synergizes with Obatoclax or JAK2 inhibitors in MM; enhances apoptosis with carfilzomib/gemcitabine/vincristine/etoposide in B-cell lymphoma; synergizes with palbociclib suppresses cell proliferation and induces cell death (CCA); Co-treatment with paclitaxel, taxinol, or ABT-263 (TNBC)	Phase I/II (NCT01098838, NCT01617668, NCT01955434, NCT02098161)	Well tolerated with Pharmacodynamics activity; limited efficacy as monotherapy; Synergy with paclitaxel increased pCR with manageable toxicity; objective responses in myelofibrosis with reduced cIAP levels	[239–263]
	Xevinapant (SM-406/AT-406/Debio 1143)	XIAP, cIAP1/2	Suppresses XIAP/cIAP1-mediated survival; activates caspase-3/8/9–dependent apoptosis; modulates NF-κB and enhances TNF-α signaling; potentiates chemo-, radio-, and immunotherapy responses	Induces apoptosis in breast, ovarian, pancreatic, and colorectal cancers; reverses carboplatin resistance (ovarian); synergy with Bcl-2 inhibition (ABT-737/ABT-199) augments efficacy; enhances apoptosis with rocaglamide and blue LED; synergizes with TRAIL (NPC); synergizes with ionizing radiation (HNSCC)	Phase II/III (NCT02022098, NCT04122625, NCT03270176, NCT03871959, NCT04459715, NCT04962724, NCT05519540, NCT01078649, NCT05724602, NCT06145412, NCT06110195)	In HPV-negative HNSCC, cisplatin-chemoradiotherapy markedly improves OS, PFS, and locoregional control; manageable epithelial toxicity (mucositis/dysphagia); xevinapant vs placebo plus CRT did not improve EFS; safe with PD-1/PD-L1 inhibitors; synergizes with pembrolizumab well tolerated but limited efficacy	[264–281]
	BV6	cIAP1/2, XIAP	Induces degradation of cIAP1/2; abolishes IAP-mediated caspase inhibition; promotes NF-κB activation; triggers TNFα-dependent apoptosis and enhances radiosensitivity	Promotes apoptosis and radiosensitization in NSCLC (HCC193, H460); exerts cytotoxicity across multiple HNSCC lines; synergizes with FasL; augments cisplatin-induced apoptosis and overcomes chemoresistance in tongue carcinoma; synergizes with ALM301 and overcome resistance to AKT inhibition and platinum based chemotherapy; enhances susceptibility to NK cell cytotoxicity in pediatric rhabdomyosarcoma	Preclinical	Not yet in clinical trials	[282–287]
	Birinapant (TL32711)	XIAP, cIAP1/2, ML-IAP	Promotes proteasomal degradation of cIAP1/2; activates RIPK1–caspase-8 apoptosis; suppresses TNFα-induced NF-κB signaling	Monotherapy efficacy in 16% of tumor models; synergizes with chemo/radiotherapy, TNFα/TRAIL, and immunotherapy; enhances gemcitabine-induced apoptosis (TNBC); sensitizes CRC to chemotherapy and iontotherapy; restores TRAIL sensitivity (glioblastoma); radiosensitizes NSCLC; potentiates irinotecan- or bortezomib-induced apoptosis (MM); synergy with TNFα and KIT inhibitors (imatinib, regorafenib) in melanoma and GIST; synergizes with WEE1 sensitizes TNFα- and radiation-induced cell death in HNSCC	Phase I/II (NCT01940172, NCT01828346, NCT01940172, NCT01188499, NCT02587962, NCT03803774)	Synergy with conatumumab (recurrent ovarian cancer) and PD-1/CTLA-4 inhibitors (glioblastoma)	[288–301]
	SM-164	XIAP, cIAP1/2	Antagonizes IAP-mediated apoptosis inhibition; activates caspase-3/-9 and promotes PARP cleavage; suppresses AKT signaling	Exhibits pro-apoptotic activity in breast (MDA-MB-231), leukemia (HL-60), ovarian (SK-OV-3), and melanoma (MALME-3 M) cells; suppresses metastasis and eradicates early lesions in MDA-MB-231 xenografts; enhances radiosensitivity (HNSCC); co-treatment with doxorubicin enhances caspase activation and chemosensitivity (HCC)	Preclinical	Not yet in clinical trials	[302–305]
	ASTX660 (Tolinapant)	XIAP, cIAP1/2	Induces apoptosis and necroptosis; enhances TNFα/TRAIL/FasL-mediated cytotoxicity; augments tumor–immune interactions and TIL cytotoxicity; promotes immunogenic cell death (ICD)	Induces necroptosis in apoptosis-resistant bladder cancer; limited monotherapy efficacy in HNSCC lines; synergizes with radiotherapy, anti–PD-1 therapy and TRAIL/TNFα (HPV – and HPV+ HNSCC models); enhances TNFα/TRAIL/FasL/cisplatin sensitivity (MOC1/MOC22); promotes ICD; increases CD8^+^ T-cell and dendritic cell activity	Phase I/II (NCT05082259, NCT05912075, NCT05245682, NCT06393751, NCT05403450, NCT06590558)	Well tolerated; preliminary safety acceptable	[306–312]
	APG-1387	XIAP, cIAP1/2	Promotes degradation of IAPs; activates RIPK1 and caspase-8; induces TNFα-dependent apoptosis and RIPK1-driven necroptosis; enhances IL-12 secretion and immune activation	Reduces IAP levels but limited cytotoxicity alone, synergizes with TNFα or TRAIL to induce caspase-dependent apoptosis and RIPK1-driven necroptosis (HCC); drives caspase-8/FADD/RIP1 complex assembly, enhances IL-12 and T-cell activity, and synergy TRAIL co-treatment selectively targets CSCs and reduces tumor stemness (ovarian cancer); synergy with anti–PD-1 therapy; increases chemosensitivity(cisplatin/5-FU, NPC); synergizes with PARP inhibitors (olaparib, BRCA1/2 tumors) and MEK inhibitors (trametinib, KRAS-mutant pancreatic cancer)	Phase I/II (NCT03386526, NCT04568265, NCT04284488, NCT04643405)	Early safety acceptable; Preliminary safety manageable	[271, 313–319]
Antisense oligonucleotide	LY2181308	Survivin mRNA	Downregulates Survivin expression; induces caspase-3–dependent apoptosis; causes G2/M arrest and multinucleation; enhances chemosensitivity and radiosensitivity	Induces apoptosis and cell-cycle arrest in HCC; enhances radiosensitivity and synergizes with gemcitabine, paclitaxel, and docetaxel (CRC)	Phase I/II (NCT00415155, NCT00642018, NCT01107444, NCT00620321)	Well tolerated with favorable safety; limited monotherapy efficacy; combination with cytarabine/idarubicin in AML showed synergistic activity without dose-limiting toxicity	[320–322]
	AEG 35156	XIAP	Degrades XIAP expression in a dose-dependent manner; inhibit tumor growth; enhances chemosensitivity	Reduces tumor volume by 40% alone, in combination with docetaxel achieves 77% reduction within 3 weeks (NSCLC); Strong synergy with standard chemotherapeutics	Phase I/II (NCT00357747, NCT00372736, NCT00363974)	Well tolerated and Safe	[182, 323]
	EZN-3042	Survivin exon 4	Binds to the stop codon region within Survivin exon 4; suppresses Survivin and Bcl-2 expression; enhances chemosensitivity	Enhances paclitaxel sensitivity (prostate cancer); inhibits tumor growth by 40%, and with paclitaxel achieves > 80% suppression (Lung cancer)	Phase I (NCT01186328)	Dose-limiting toxicities and adverse drug-drug interactions	[324, 325]
Ribozymes	RZ-1/RZ-2	Survivin mRNA	Sequence-specific endonucleolytic cleavage of Survivin mRNA	Induces apoptosis and enhances chemosensitivity under stress (breast cancer); increases cisplatin sensitivity and suppresses tumor growth (androgen-independent prostate cancer)	Preclinical	Not yet in clinical trials; Misfolding and RNA degradation when vectorized	[326–329]
Small-molecule inhibitors	YM155	Survivin promoter	Targets Survivin; activates PUMA-mediated and caspase-3–dependent apoptosis; inhibits Akt/mTOR and upregulates Beclin-1 to promote apoptosis and autophagy	Induces apoptosis and suppresses growth (HRPC); enhances apoptosis/autophagy (HNSCC); induces dose-dependent apoptosis (esophageal cancer); synergy with decitabine (leukemia)	Phase II	Well tolerated with favorable safety; limited clinical efficacy	[330–334]
	EM-1421 (Terameprocol)	Survivin, Cdc2	Inhibits Sp1-dependent transcription of Survivin and Cdc2; activates mitochondrial apoptosis; induces G2/M arrest; enhances radiosensitivity	Broad tumor suppression across multiple xenograft models; enhances radiosensitivity in NSCLC	Phase I/II (NCT00664677, NCT02575794, NCT00154089, NCT00259818)	Good tolerability and partial responses in CML/AML. No objective responses in glioma	[335–338]
	FL-118	Survivin, Mcl-1, XIAP, cIAP2	Inhibits Survivin promoter activity and induces DNA damage	Strong antitumor efficacy in colorectal and head-neck cancer	Preclinical	Not yet in clinical trials	[335, 339]
Hsp90 inhibitors	Shepherdin	Hsp90–Survivin complex	Disrupts Hsp90–Survivin interaction; induces mitochondrial dysfunction and mitotic catastrophe, and caspase-dependent apoptosis	Triggers apoptosis across multiple cancer cell lines; causes tumor regression in breast and prostate cancer xenografts with minimal toxicity to normal fibroblasts	Preclinical	Not yet in clinical trials	[340, 341]
	AICAR	Hsp90 client proteins (including Survivin)	Disrupts Hsp90–client proteins interactions; promotes ubiquitin-mediated degradation of Survivin and other client proteins	Antiproliferative activity in prostate cancer, osteosarcoma, and ALL; negligible toxicity to normal cells	Preclinical	Not yet in clinical trials	[342, 343]
	17-DMAG	Survivin–Hsp90 complex	Disrupts Hsp90–Survivin interaction; promotes Survivin degradation and apoptosis	Induces apoptosis, and proliferation arrest in HCC models	Preclinical	Not yet in clinical trials	[344]
CDKs inhibitors	Flavopiridol, Purvalanol A	Survivin (Thr34 phosphorylation)	Inhibit CDK1–cyclin B1–mediated phosphorylation of Survivin at Thr34 during mitosis; enhance paclitaxel-induced mitotic arrest and apoptosis	Overcoming paclitaxel resistance; exhibit potent antitumor efficacy in preclinical models	Preclinical	Not yet in clinical trials	[345]
HDAC inhibitors	Clamydocin	Survivin (epigenetic)	Induces hyperacetylation of histones H3 and H4; activates caspase-3; upregulates p21 (CDKN1A)	Causes cell-cycle arrest and induces apoptosis	Preclinical	Not yet in clinical trials	[346]
	Dacinostat (LAQ824)	Survivin (epigenetic)	downregulates Survivin expression; induces p21 upregulation; triggers apoptosis	Promotes apoptosis and growth inhibition in diverse cancer models	Preclinical	Not yet in clinical trials	[347]
	Belinostat (Beleodaq)	Survivin (epigenetic)	Degrades TGF-β-dependent Survivin. expression	Induces apoptosis in tumor models	Preclinical	Not yet in clinical trials	[348]
	Apigenin	Multiple IAPs (including Survivin)	Modulates cell-cycle genes; induces chromatin remodeling and caspase-dependent apoptosis	Promotes growth inhibition and induces apoptosis in prostate cancer	Preclinical	Not yet in clinical trials	[349]
Vaccine therapy	Survivin-derived epitope vaccines (Survivin-2B80-88, SVN53-67/M57-KLH)	Survivin (antigen)	Survivin as a TAA; enhances dendritic cell, NK cell, and CTL-mediated immune responses	Survivin peptides induce strong CD8 T-cell activation and tumor suppression in colon cancer, lung cancer, neuroblastoma, and prostate cancer; synergizes with Survivin mRNA and the STAT3 inhibitor stattic enhanced antitumor activity and improved survival (colon cancer); peptide mimetics (SVN53–67/M57-KLH) elicit systemic antitumor immunity and enhance adoptive immunotherapy	Phase I	Well-tolerated with no dose-limiting toxicities	[350–355]

Abbreviations: AML, acute myeloid leukemia; ALL, acute lymphoblastic leukemia; BIR, baculoviral IAP repeat; CDK, cyclin-dependent kinase; Cdc2, cell division control protein 2; cIAP1/2, cellular inhibitor of apoptosis protein 1/2; HCC, hepatocellular carcinoma; HNSCC, head and neck squamous cell carcinoma; Hsp90, heat shock protein 90; IAP, inhibitor of apoptosis protein; ICD, immunogenic cell death; MM, multiple myeloma; NF-κB, nuclear factor kappa-light-chain-enhancer of activated B cells; NSCLC, non-small cell lung cancer; OS, overall survival; OSCC, oral squamous cell carcinoma; PFS, progression-free survival; RIP1, receptor-interacting protein kinase 1; Smac, second mitochondria-derived activator of caspases; TAA, tumor-associated antigen; TNFα, tumor necrosis factor-alpha; TRAIL, TNF-related apoptosis-inducing ligand; XIAP, X-linked inhibitor of apoptosis protein

**Table 3:** Clinical trials of targeting IAPs therapies

Category	Identifier/reference	Indication	Phase/status	Trial start date	Outcome measure/result	Reference	
**GDC-0152** **(RG7419)**	NCT00977067	Locally advanced or metastatic tumors	Phase I: Terminated	June 2007	Limited objective responses		
**LCL161**	NCT01098838	Advanced solid tumors	Phase I: completed	November 2008	Well tolerated on cIAP1 degradation; no objective response observed		[356]
	NCT01240655	Advanced solid tumors	Phase Ib: completed	April 2011	Combination with paclitaxel was safe and pharmacodynamically active; limited clinical efficacy observed		[357]
	NCT01617668	TNBC	Phase II: completed	August 2012	Effective with paclitaxel (gene-positive patients), significant toxicity		[358]
	NCT01968915	Advanced solid tumors (Japan)	Phase I: completed	November 2013	manageable toxicity and pharmacodynamic target engagement without objective responses		
	NCT01955434	Relapsed/refractory multiple myeloma	Phase II: completed	November 2013	No objective responses with monotherapy; combination with cyclophosphamide yielded modest efficacy with manageable hematologic toxicity		
	NCT01934634	Metastatic pancreatic cancer	Phase I: Unknown status	March 2014	Combination with gemcitabine in pancreatic cancer showed acceptable safety but no meaningful efficacy		
	NCT02098161	PMF, post-PV MF, and post-ET MF	Phase II: Completed	December 2014	Combination with cyclophosphamide and bortezomib showed limited clinical benefit with hematologic toxicity		
	NCT02649673	Relapsed/refractory SCLC/ovarian cancer	Phase Ib: Terminated	March 2016	Dose-limiting toxicity but limited antitumor activity		
	NCT02890069	Advanced/metastatic NSCLC, colorectal, TNBC	Phase Ib: Completed	October 2016	Manageable safety and early disease control in some patients		
	NCT03111992	Relapsed/refractory multiple myeloma	Phase I/IIb: Completed	December 2017	Partial responses with acceptable tolerability		
**Debio 1143 (AT-406)**	NCT01078649	Advanced solid tumors/lymphomas	Phase I: completed	March 2010	Well tolerated with cIAP1 degradation; limited antitumor activity		[359]
	NCT01930292	Squamous NSCLC/Platinum-refractory ovarian cancer/TNBC	Phase I: terminated	April 2013	Combination with carboplatin and paclitaxel feasible; partial responses observed but dose-limiting toxicity restricted escalation		
	NCT02202098	Stage III/IV HNSCC	Phase I/II: completed	October 2013	Combination with chemo-radiotherapy improved local control with acceptable tolerability		
	NCT03270176	Advanced solid tumors/Stage IIIb/IV NSCLC after platinum-based treatment	Phase Ib: completed	October 2017	Manageable safety and preliminary disease control with Debio 1143		
	NCT03871959	advanced/metastatic PDAC or CRC	Phase I: completed	September 2019	Combination with pembrolizumab showed good tolerability; limited antitumor activity		[275]
	NCT04122625	advanced solid tumors/Stage IIIb/IV NSCLC after platinum-based treatment failed to standard treatments	Phase I/II: completed	April 2019	Combination with nivolumab showed well tolerated with early signals of response		
	NCT04459715	LA-SCCHN	Phase III: terminated	August 2020	Combination with CRT showed favorable safety but failed primary endpoint (EFS)		[276]
	NCT05386550	LA SCCHN ineligible to receive cisplatin-based chemoradiation concurrently	Phase III: terminated	October 2022	Ongoing, no efficacy data yet		
	NCT05519540	Healthy East Asian Participants	Phase I: completed	September 2022	PK/PD consistency and tolerability		
	NCT06056310	Unresected LA SCCHN (HyperlynX)	Phase I: terminated	January 2024	Combination with CRT showed acceptable safety profile.		
	NCT06084845	Head and Neck Cancer	Phase II: withdrawn	April 2024	Ongoing trial of xevinapant combinations in solid tumors; preliminary safety acceptable		
	NCT06463184	rHGG	Phase I: withdrawn	July 2024	Recruiting early-phase study assessing novel Debio 1143 combinations		
**Birinapant (TL32711)**	NCT00993239	Refractory solid tumors/lymphomas	Phase I: completed	November 2009	Well tolerated; demonstrated on-target cIAP1 degradation with limited antitumor activity		[360]
	NCT01188499	Advanced/metastatic solid tumors	Phase Ib/IIa: completed	October 2010	Safe in combination with chemotherapy; promising clinical activity		[361]
	NCT01486784	AML/ALL/MDS	Phase I/II: terminated	November 2011	Limited single-agent efficacy with manageable hematologic toxicity		
	NCT01573780	Advanced Solid Tumors	Phase I: terminated	April 2012	Combination with docetaxel was feasible; dose-limiting neutropenia observed, limited efficacy		
	NCT01681368	Advanced/metastatic ovarian cancer	Phase II: terminated (no clinical benefit)	August 2012	Suppression of cIAP1; no clinical activity		[362]
	NCT01828346	MDS	Phase I/II: completed	June 2013	Combination with radiotherapy showed acceptable safety; efficacy not established		
	NCT01940172	Relapsed ovarian cancer	Phase Ib: completed	November 2013	Combination with conatumumab was well tolerated; limited objective responses		[363]
	NCT02147873	Myelomonocytic leukemia/MDS	Phase II: terminated (inefficacy)	June 2014	Combination with azacitidine caused significant hematologic toxicity; minimal efficacy observed		[364]
	NCT02288208	Chronic Hepatitis B	Phase I: terminated	November 2014	Combination with pembrolizumab was feasible; suggested immune-mediated tumor necrosis		
	NCT02756130	High-grade serous ovarian/endometrial cancer	Phase II: withdrawn	August 2018	Results pending		
	NCT02587962	Advanced/metastatic solid tumors	Phase I/II: recruiting	August 2017	Combination with pembrolizumab showed acceptable safety and early disease control		
	NCT03803774	locoregionally recurrent HNSCC	Phase I: Active, not recruiting	September 2019	Combination with radiation demonstrated manageable toxicity		
**ASTX660** **(Tolinapant)**	NCT02503423	Advanced solid tumors/lymphomas	Phase I/II: Active, not recruiting	July 2015	Well tolerated; dose-limiting toxicities included fatigue and nausea; preliminary disease stabilization observed		
	NCT04155580	Relapsed/Refractory AML	Phase I: terminated	June 2020	Combination with ASTX727 (cedazuridine/decitabine) was tolerable; early data showed hematologic responses		
	NCT04362007	Relapsed or Refractory T-Cell Lymphoma	Phase I/II: terminated	July 2020	Early-phase study ongoing, no efficacy data yet		
	NCT05082259	immune-refractory cancers, triple negative breast cancer (TNBC), cervical cancer, and glioblastoma.	Phase I: recruiting	March 2022	Ongoing, no efficacy data yet		
	NCT05245682	Locally Advanced Head and Neck Cancer	Early Phase I: Active, not recruiting	February 2022	Combination with decitabine ongoing; preliminary safety acceptable		
	NCT05403450	Relapsed/Refractory Peripheral T-cell Lymphoma (R/R PTCL)	Phase I/II: Active, not recruiting	June 2022	Combination with radiotherapy under investigation; early safety profile acceptable, results pending		
	NCT05912075	Rectum Cancer (PRAAR1)	Phase I: recruiting	December 2023	Combination with venetoclax and/or azacitidine ongoing, no efficacy data reported		
	NCT06393751	Recurrent Ovarian Cancer	Phase I/II: withdrawn	October 2025	Combination with checkpoint inhibitor; preliminary results pending		
	NCT06590558	Advanced TNBC	Phase I: withdrawn	August 2025	Newly initiated trial; no data yet		
**APG-1387**	NCT03386526	Advanced Solid Tumors or Hematologic Malignancies	Phase I: completed	November 2017	Study terminated early with limited efficacy data available		
	NCT04284488	Solid Tumors	Phase I/II: Recruiting	April 2020	Combination with checkpoint blockade; early safety acceptable, clinical efficacy not reported		
	NCT04643405	advanced pancreatic adenocarcinoma	Phase I/II: Recruiting	March 2021	Preliminary safety manageable, results pending		
**LY2181308**	NCT00620321	Relapsed/refractory AML	Phase II: completed	March 2008	Monotherapy was well tolerated but produced no confirmed objective responses in advanced solid tumors		
	NCT00642018	Castration-resistant prostate cancer	Phase II: completed	March 2008	Well tolerated but failed to improve clinical outcomes		
	NCT01107444	Stage IIIb/IV NSCLC	Phase II: completed	May 2010	Combination with chemotherapy showed pharmacodynamic Survivin suppression but was limited by toxicity and minimal efficacy		
	NCT00415155	Advanced Hepatocellular Carcinoma	Phase I/II: withdrawn	August 2025	Combined with docetaxel was safe and achieved partial responses		
**AEG 35156**	NCT00357747	Solid Tumors	Phase I: completed	June 2005	Combination with docetaxel: dose-escalation defined tolerable dose; no confirmed objective responses reported		
	NCT00363974	Refractory/relapsed AML	Phase I/II: completed	October 2005	Safe and XIAP knock-down achieved and improved response rate		
	NCT00372736	Locally Advanced, Metastatic, or Recurrent Solid Tumors	Phase I: completed	July 2006	Safe in combination with docetaxel; no posted efficacy results		
	NCT00385775	Advanced Cancers	Phase I: terminated	June 2006	No efficacy results		
	NCT00557596	Advanced pancreatic cancer	Phase I: terminated	September 2007	Safe in gemcitabine, failed to show additional clinical benefit		
	NCT00558922	Stage IIIb/IV NSCLC	Phase I/II: terminated (neurotoxicity)	September 2007	Safe in combination with carboplatin and paclitaxel; progression-free survival		
	NCT00558545	Advanced breast cancer	Phase I/II: terminated	November 2007	Safe in paclitaxel; progression-free survival		
	NCT00768339	Relapsed/refractory CLL/indolent B-cell lymphoma	Phase I/II: terminated (slow recruitment)	September 2008	Safe tolerated dose; Objective tumor response		
	NCT00882869	HCC	Phase I/II: completed	March 2009	Well tolerated with sorafenib; enhanced anti-tumor activity		
	NCT01018069	Primary refractory AML	Phase II: terminated	November 2009	Well tolerated in combination with cytarabine and idarubicin but failed to improve remission rate		
**EZN-3042 (Survivin mRNA antagonist)**	NCT01186328	Relapsed ALL (pediatric)	Phase I: terminated	August 2010	Significant toxicity		[365]
**YM155 (small molecule Survivin inhibitor)**	NCT00281541	Unresectable Stage III or Metastatic Stage IV Melanoma	Phase II: completed	November 2005	Well tolerated but primary efficacy endpoint not met		[366]
	NCT00328588	Stage IIIb/IV NSCLC	Phase II: completed	December 2006	Safe and tolerable; modest single-agent activity		[367]
	NCT00257478	Hormone Refractory Prostate Cancer (HRPC)	Phase II: completed	March 2007	Achieved partial response		[368]
	NCT00514267	Advanced hormone refractory prostate cancer/solid tumors	Phase I/II: completed	May 2007	Safety and efficacy		
	NCT00498914	Refractory DLBCL	Phase II: terminated (futility)	June 2007	No significant response rate		[369]
	NCT01023386	Advanced Cancer	Phase I: completed	November 2009	No objective responses reported; pharmacologic exposures achieved but biomarker modulation not clearly correlated with clinical benefit.		
	NCT01007292	Relapsed B-cell NHL	Phase II: completed	November 2009	Combination with rituximab with tolerable and durable response		[370]
	NCT01009775	Stage III/IV melanoma	Phase II: completed	November 2009	Combination with rituximab with well-tolerated but not improved PFS		[371]
	NCT01038804	Her-2 negative metastatic breast cancer	Phase II: completed	December 2009	Combination with docetaxel with well-tolerated but endpoints not met		[372]
	NCT01100931	Solid tumors/NSCLC	Phase I/II: completed	February 2010	Combination with carboplatin and paclitaxel, but not improvement in response		[373]
	NCT05263583	High-grade B-cell Lymphoma	Phase II: Active, not recruiting	December 2022	Achieved notable activity, with short-to-moderate response duration		[374]
**EM-1421** **(transcription inhibitor)**	NCT00154089	Cervical Intraepithelial Neoplasia	Phase I/II: completed	November 2004	Good safety profile but no efficacy data report		[375]
	NCT00259818	Recurrent or Refractory Solid Tumors	Phase I: completed	December 2005	Safety/tolerability up to highest planned dose, minimal objective responses reported		
	NCT00404248	Recurrent high-grade glioma	Phase I/II: completed	January 2007	Safe with modest anti-tumor activity		[376]
	NCT00664586	Refractory solid tumors/lymphomas	Phase I: terminated (financial)	May 2007	Max tolerated dose; dose-limiting toxicity, Anti-tumor activity		
	NCT00664677	Leukemias	Phase I: terminated (financial)	August 2007	Max tolerated dose; dose-limiting toxicity, Anti-tumor activity		
	NCT02575794	Recurrent high-grade glioma	Phase I: suspended (drug production issues)	August 2018	Safe but showed poor bioavailability and minimal activity		[375]
**SVN53-67/M57-KLH**	NCT01250470	Malignant Glioma	Phase I: completed	September 2012	Well-tolerated with no dose-limiting toxicities; suggested immunogenicity but efficacy unconfirmed		[377]
	NCT02455557	Newly Diagnosed Glioblastoma	Phase II: Active, not recruiting	May 2015	Well tolerated; high antibody titers correlated with improved survival		
	NCT02334865	Newly Diagnosed Multiple Myeloma Receiving Lenalidomide Maintenance Therapy	Phase I: Active, not recruiting	April 2017	Well-tolerated; no dose-limiting toxicities reported; efficacy/immunogenicity results not yet published		
	NCT03879694	Metastatic Neuroendocrine Tumors	Phase I: completed	June 2019	No results posted		
	NCT05163080	Newly Diagnosed Glioblastoma	Phase II: Active, not recruiting	November 2021	Ongoing		
	NCT06202066	Metastatic Neuroendocrine Carcinomas	Phase II: Not yet recruiting	November 2025	Not yet recruiting		
	NCT07169617	Lung Cancer Prevention	Phase II: Not yet recruiting	February 2026	Not yet recruiting		

Abbreviations: AML, acute myeloid leukemia; ALL, acute lymphoblastic leukemia; ASO, antisense oligonucleot; BIRC2/3, baculoviral IAP repeat containing 2/3 (cIAP1/2); BIRC5 (Survivin), baculoviral IAP repeat containing 5; BIR, baculoviral IAP repeat domain; XIAP (BIRC4), X-linked inhibitor of apoptosis protein; BCL, B-cell lymphoma; cIAP, cellular inhibitor of apoptosis protein; CIN, cervical intraepithelial neoplasia; CLL, chronic lymphocytic leukemia; CRC, colorectal cancer; CRT, chemoradiotherapy; DLBCL, diffuse large B-cell lymphoma; EFS, event-free survival; ETMF, essential thrombocythemia-related myelofibrosis; GBM, glioblastoma multiforme; HCC, hepatocellular carcinoma; HNSCC, head and neck squamous cell carcinoma; HRPC, hormone-refractory prostate cancer; IAP, inhibitor of apoptosis protein; LA-SCCHN, locally advanced squamous cell carcinoma of the head and neck; MF (PMF/post-PV/post-ET), myelofibrosis (primary/post-polycythemia vera/post-essential thrombocythemia); MDS, myelodysplastic syndrome; NHL, non-Hodgkin lymphoma; NSCLC, non-small-cell lung cancer; OS, overall survival; PDAC, pancreatic ductal adenocarcinoma; PD-1, programmed cell death protein 1; PFS, progression-free survival; PK/PD, pharmacokinetics/pharmacodynamics; PTCL, peripheral T-cell lymphoma; R/R, relapsed/refractory; rHGG, recurrent high-grade glioma; SCLC, small-cell lung cancer; SMAC, second mitochondria-derived activator of caspase; SMAC mimetic, small-molecule IAP antagonist mimicking endogenous SMAC/DIABLO; TNBC, triple-negative breast cancer

### SMAC and Smac mimetics

Second mitochondria-derived activator of caspases (SMAC/DIABLO) is the best-characterized endogenous antagonist of IAPs [378]. Upon apoptotic stimulation, SMAC—originally synthesized as a mitochondrial precursor—is proteolytically processed and released into the cytosol. Its N-terminal IAP-binding motif (IBM) directly engages the BIR domains of XIAP, cIAP1, and cIAP2, thereby neutralizing their caspase-inhibitory activity and promoting activation of caspases-3, −7, and −9, which ultimately execute apoptosis [379]. Functionally, Smac overexpression enhances TRAIL-induced death, suppresses proliferation of leukemia cells, and restores apoptotic sensitivity even in cell lines with low apoptotic protease activating factor-1 (Apaf-1) expression, thus activating both intrinsic and extrinsic death pathways [380].

Building on the structural basis of Smac-IAP interaction, researchers have developed small-molecule IAP antagonists, collectively known as Smac mimetics (SMs), which successfully restore Smac/DIABLO function by imitating its crucial N-terminal tetrapeptide AVPI motif [381]. SMs are categorized into monovalent and divalent compounds depending on their structural configuration [379] Divalent SMs, comprising of two monomeric units linked by a chemical linker, exhibit higher IAP affinity, enhanced antagonistic potency, and stronger pro-apoptotic activity than monovalent counterparts, although their in vivo bioavailability remains limited [382, 383. P]reclinical studies have demonstrated that peptidic analogues such as Smac-4 and Smac-7 significantly enhance chemotherapy- and immunotherapy-induced apoptosis in leukemia models [89]. Moreover, SMs have shown broad chemosensitizing potential across multiple tumor types—including breast cancer, multiple myeloma, malignant glioma, and non–small cell lung cancer (NSCLC: H460)—by overcoming resistance to conventional cytotoxic agents and augmenting TRAIL-induced apoptosis [229].

GDC-0152/RG7419, developed by Genentech, was the first Smac mimetic to enter clinical trials [381]. It functions as a pan-IAP antagonist, binding with high affinity to BIR3 domains of cIAP1, cIAP2, XIAP, and Livin, while showing comparatively lower affinity for the BIR2 domains of cIAP1 and cIAP2 [230]. Mechanistically, GDC-0152 promotes degradation of cIAPs, activates caspases 3 and 7, and inhibits the PI3K/AKT pathway, leading to apoptosis in multiple tumor cells, including melanoma, glioblastoma, breast cancer, and osteosarcoma models [231, 232]. It also suppresses ANGPTL2-driven malignancy and exerts synergistic effects with TNF-α released from tumor-associated immune cells in osteosarcoma [233–235]. In vivo, GDC-0152 demonstrated dose-dependent efficacy, delaying tumor growth and promoting apoptosis in U87MG neuroblastoma and MDA-MB-231 breast cancer xenograft models [231, 236]. Under hypoxic conditions, an important cause of therapeutic resistance, GDC-0152 also induced apoptosis in stem-like glioblastoma cells, reducing their proliferative capacity [234, 237, 238]. Despite encouraging preclinical results, its Phase I clinical study (NCT00977067) in patients with advanced or metastatic malignancies was prematurely terminated due to insufficient objective responses [230].

LCL161, developed by Novartis, is a monovalent, orally bioavailable Smac mimetic that binds with high affinity to BIR3 domains of cIAP1 and cIAP2 and with lower affinity for XIAP-BIR3 [239]. Mechanistically, LCL161 disrupts the interaction between IAPs and caspases, inducing proteasomal degradation of cIAP1/2 and altering NF-κB signaling, which enhances TNF-α production, T-cell activation, and cytokine secretion [240, 241]. It also triggers apoptosis and necroptosis through the RIP1/RIP3/MLKL pathway. In addition, LCL161 has been identified as a regulator of ABCB1/MDR1-ATPase and modulator of BIRC5/Survivin expression while suppressing the PI3K/AKT pathway and mitochondrial membrane potential in hepatocellular carcinoma cells [241]. Preclinical studies have confirmed the broad pro-apoptotic and antiproliferative potential of LCL161 across multiple tumor types. When combined with paclitaxel in non-small cell lung cancer (NSCLC) models, it promotes cIAP1/2 degradation, activates caspase-8-dependent apoptosis, and upregulates TNFα expression, thereby enhancing chemosensitivity [242]. In head and neck squamous cell carcinoma (HNSCC), LCL161 alone has little limited cytotoxicity but markedly enhances radiation- and FasL-induced apoptosis by promoting DNA damage and caspase activation [243, 382]. Furthermore, in HPV-related HNSCC, LCL161 preferentially sensitizes tumors to radiotherapy via cIAP1 inhibition [244]. In hepatocellular carcinoma (HCC), co-targeting Bcl-2 with the novel inhibitor SC-2001 enhances Smac-mimetic efficacy and overcomes LCL161 resistance, establishing a promising anti-IAP combination therapy [245]. Similarly, in esophageal squamous cell carcinoma (ESCC), LCL161 acts as a radiosensitizer by promoting caspase-8 activation and apoptosis [246], and suppresses ECA109 cell proliferation and reduces XIAP expression in a dose- and time-dependent manner [247]. The compound also exerts immune-stimulatory and oncolytic synergy: combined treatment with VSVΔ51-GFP reduces tumor volume, prolongs survival, and enhances CD8^+^ T-cell–mediated cytotoxicity, and it shows a synergistic effect without systemic toxicity with AAVP-TNF-α [248–250]. Combinations with KIT inhibitors (imatinib, regorafenib), proteasome inhibitors (carfilzomib), or chemotherapeutics (rituximab, gemcitabine, vinorelbine, etoposide) have shown synergistic anti-tumor activity in gastrointestinal cancers [251]. In hematologic malignancies, particularly multiple myeloma (MM), LCL161 acts synergistically with the Bcl-2 inhibitor Obatoclax (OBX) or JAK2 inhibitors and enhances cytotoxicity through IAPs inhibition, caspases activation, and upregulating of pro-apoptotic proteins (Bid, Bim, Puma, and Noxa) [252]. In lymphoma models, LCL161 (10 µM) alone shows minimal activity but significantly potentiates the effects of carfilzomib (5 nM), reducing IAP expression (especially cIAP1, Livin, and Bruce) and inducing apoptosis in rituximab-resistant B-cell lymphoma, though providing no additional survival benefit in rituximab-sensitive xenografts [253, 254]. It also synergizes with gemcitabine, carfilzomib, or vincristine/etoposide, improving anti-tumor efficacy and extending survival in Raji 4RH xenograft models [254]. In Cholangiocarcinoma (CCA), combined inhibition of CDK4/6 with palbociclib and cIAP1/2 with LCL161 effectively suppresses cell proliferation and induces cell death, while exhibiting minimal effects on normal cholangiocytes and peripheral blood mononuclear cells (PBMCs) [255]. However, clinical efficacy remains limited: a Phase II trial (NCT01955434), reported no objective responses with LCL161 monotherapy, compared with a 17.4% response rate for LCL161 plus cyclophosphamide [256]. LCL161 also demonstrates potent anti-tumor effects in solid tumors. In breast cancer, it induces cIAP1 degradation and activates caspase-dependent apoptosis and necrosis in both MDA-MB-231 and MCF-7 cells, triggering multiple programmed cell death pathways. Co-treatment with paclitaxel, taxinol, or ABT-263 (Bcl-2 inhibitor) further enhances apoptosis, suggesting therapeutic potential in breast cancers [241, 257–260]. Moreover, targeting cIAP1 with LCL161 restores caspase-8 activation and overcomes PAR-4–deficiency–induced chemoresistance in TNBC, underscoring its translational relevance [260, 261]. Clinically, Phase I and II trials have established favorable tolerability and a weekly 1800 mg oral dose as the recommended regimen (NCT01098838), with significant pharmacodynamic activity [253]. In triple-negative breast cancer (NCT01617668), combination with paclitaxel increased pathologic complete response (pCR) rates with manageable toxicity [252, 262]. Additionally, in myelofibrosis (NCT02098161) reported objective responses in 30% of high-risk patients, all showing reduced cIAP levels, suggesting potential use in elderly, thrombocytopenic, or JAK inhibitor-refractory cases [263].

Xevinapant (SM-406/AT-406/Debio 1143), an isovaleryl analogue and orally monovalent Smac mimetic developed by Ascenta, exhibits strong affinity for XIAP-BIR3, cIAP1, and cIAP2 [264]. Mechanistically, Xevinapant promotes caspase-3/8/9–dependent apoptosis and suppresses survival in multiple malignancies, including breast, ovarian, and colorectal cancers [265]. In preclinical models, Xevinapant induces caspase-8-dependent apoptosis in MDA-MB-231 breast cancer xenografts [266], and reverses carboplatin resistance in ovarian cancer, enhancing and restoring carboplatin-induced cytotoxicity both in vitro and in vivo [267, 268]. In pancreatic cancer, Xevinapant markedly inhibits tumor proliferation and induces apoptosis by downregulating XIAP and cIAP1 without affecting Bcl-2; conversely, inhibition of Bcl-2 with ABT-737 or shRNA significantly enhances its cytotoxic efficacy, highlighting Bcl-2 as a key determinant of resistance and a promising target for combination regimens [269, 270]. Similarly, in colorectal adenocarcinoma models, dual inhibition strategies combining Xevinapant with a Bcl-2 inhibitor (ABT-199) or a BRAF inhibitor markedly reduce tumor viability, and blue LED–based cotreatments with rocaglamide further amplify apoptosis, suppress autophagy, and increase reactive oxygen species (ROS) production [271]. In nasopharyngeal carcinoma (NPC) S18/S26 cell lines, Xevinapant combined with TRAIL promotes cIAP1 degradation and triggers apoptosis signaling [272]. Beyond chemotherapy, Xevinapant exerts synergistic interactions with radiotherapy and immune checkpoint blockade. In head and neck squamous cell carcinoma (HNSCC), Xevinapant exhibited cytostatic, cytotoxic, and radiosensitizing effects on malignant cells, while exerting minimal impact on normal tissue cells [273]. Clinically, a pivotal Phase II trial in HPV-negative HNSCC (NCT02022098), Xevinapant combined with cisplatin-based chemoradiotherapy dramatically increases OS, PFS, and locoregional control compared with chemoradiotherapy alone [274]. Although combination with radiation may increase epithelial toxicity (mucositis or dysphagia) [274], subsequent studies confirmed a favorable safety profile and pharmacologic compatibility with immune checkpoint inhibitors. Importantly, Xevinapant displays favorable immunomodulatory synergy when combined with anti–PD-1/PD-L1 therapy. In a Phase Ib/II trial (NCT04122625) for PD-1/PD-L1–refractory tumors, a recommended dose of 200 mg daily for Xevinapant in combination with nivolumab showed no dose-limiting toxicities (DLTs) or pharmacokinetic interference, supporting the clinical feasibility of IAP–immunotherapy co-targeting [253, 382]. However, in a Phase Ib/II trial, Xevinapant combined with pembrolizumab was well tolerated without unexpected adverse events, but the antitumor activity was limited (NCT03871959) [275]. Furthermore, the Phase III TrilynX trial (NCT04459715) showed no improvement in event-free survival and a higher incidence of severe adverse events with xevinapant plus chemoradiotherapy compared with placebo [276]. Pharmacokinetic assessments have also been completed in healthy East Asian males (NCT04962724, NCT05519540), advanced solid tumor/lymphoma patients (NCT01078649), and concurrent cisplatin-radiotherapy cohorts (NCT02022098) [274, 277]. Additional ongoing studies explore Xevinapant with avelumab (NCT03270176) and in combination with platinum-based chemotherapy and radiotherapy regimens in elderly or post-surgical HNSCC cohorts (NCT05724602, NCT06145412, NCT06110195) [278–281].

BV6, a divalent Smac mimetic with high affinity for cIAP1, cIAP2, and XIAP [103], promotes proteasomal degradation of cIAP1/cIAP2, thereby abolishing IAP-mediated caspase inhibition and inducing TNFα-dependent cell death [282]. In NSCLC models, BV6 markedly induces apoptosis and increases radiosensitivity in HCC193 and H460 cell lines, indicating a potent radiosensitizing effect [283]. In HNSCC, BV6 monotherapy produced broad cytotoxic effects across all ten tested cell lines, while synergistic interactions with FasL were observed in eight lines, leading to a significantly increased cytotoxicity and enhanced sensitivity to cisplatin-induced apoptosis [284]. Consistently, in tongue carcinoma models, BV6 synergizes with cisplatin to further augment apoptosis induction, even in cisplatin-resistant cells, demonstrating its potential to overcome chemoresistance via IAP inhibition [285]. Similarly, in esophageal adenocarcinoma (EAC), combined treatment with the IAP antagonist BV6 and the AKT inhibitor ALM301 demonstrated strong synergistic cytotoxicity, thereby overcoming resistance to AKT inhibition and platinum-based chemotherapy [286]. In pediatric rhabdomyosarcoma, BV6 enhances the susceptibility to NK cell–mediated cytotoxicity by promoting IAP degradation, NF-κB activation, and caspase-8–dependent cell death [287].

Birinapant (TL32711), a bivalent Smac mimetic developed by TetraLogic, functions as a potent antagonist of XIAP, cIAP1, cIAP2, and ML-IAP, promoting their proteasomal degradation and restoring apoptotic signaling [279]. At the molecular level, Birinapant binds to the BIR3 domain of cIAP1 and to the BIR domains of XIAP, cIAP2, and ML-IAP, resulting in cIAP1/2 degradation, RIPK1 activation, and subsequent caspase-8–dependent apoptosis. By eliminating TRAF2-associated cIAP1/2 complexes, it suppresses TNFα-induced NF-κB activation [288]. Preclinical screening across 111 malignant tumor models showed revealed monotherapy sensitivity in approximately 16% of tumors with confirmed single-agent efficacy in ovarian and colorectal cancer and melanoma xenografts [230, 242]. Furthermore, Birinapant displays broad synergistic potential across various therapeutic modalities, including chemoradiotherapy, TNFα/TRAIL, and immunotherapy [289]. In triple-negative breast cancer (TNBC), Birinapant enhances gemcitabine-induced apoptosis through degradation of cIAP2/XIAP and activation of the intrinsic apoptosis pathway [251, 290]. In colorectal cancer (CRC), Birinapant sensitizes tumor cells to chemotherapy-induced apoptosis, which alone is insufficient to trigger nuclear apoptotic changes but is markedly potentiated when combined with Birinapant-based iontotherapy [291]. In glioblastoma, although some resistant cell lines exhibit limited TRAIL responsiveness, combination therapy with Birinapant and ABT-199 restores apoptotic sensitivity [292]. In HNSCC, combined birinapant and G2/M checkpoint kinase WEE1 synergistically sensitizes TNFα- and radiation-induced cell death by promoting apoptosis and impairing DNA damage repair [293]. Birinapant also enhances apoptosis through cIAP1/2 depletion and caspase-3 activation, thereby exerting potent radiosensitizing effects in NSCLC [294]. When combined with irinotecan, a TNFα inducer, it markedly augments cytotoxicity and tumor regression [295], while combination with bortezomib in multiple myeloma suppresses non-canonical NF-κB signaling and promotes extrinsic apoptosis. Preclinical evidence further supports that exogenous TNFα further potentiates Birinapant-mediated cytotoxicity in melanoma [296], and synergy between Birinapant and KIT inhibitors (imatinib, regorafenib), which enhances the response in gastrointestinal stromal tumors (GIST) [290]. Clinical translation of these synergistic effects has progressed rapidly. In a Phase II trial, Birinapant combined with conatumumab (a TRAILR2 mAb) in recurrent ovarian cancer (NCT01940172) and with immune checkpoint inhibitors (anti–PD-1/CTLA-4 antibodies) in glioblastoma improved survival outcomes [297, 298]. Completed trials include combination regimens with: 5-azacitidine in myelodysplastic syndromes (NCT01828346) [299], chemotherapy (carboplatin, paclitaxel, irinotecan, docetaxel) in advanced solid tumors (NCT01188499) [300], and pembrolizumab in solid malignancies (NCT02587962) [383]. An ongoing Phase I/II study is assessing Birinapant combined with re-irradiation in recurrent HNSCC (NCT03803774) [301].

SM-164, a non-peptide divalent Smac mimetic with high affinity for XIAP and cIAPs, induces XIAP dimerization by binding to its BIR2 and BIR3 domains, leading to efficient antagonism of IAP-mediated apoptosis inhibition [302]. It exhibits dose-dependent pro-apoptotic activity across diverse cancer cell lines—including MDA-MB-231 (breast cancer), HL-60 (leukemia), SK-OV-3 (ovarian cancer), and MALME-3 M (melanoma). In breast cancer models, SM-164 not only suppresses metastatic progression but also eliminates early lesions and reduces advanced bone and lung metastasis in MDA-MB-231 xenografts, achieving complete regression without significant toxicity to normal tissue [303, 304]. In UMSSCC-1 (HNSCC), SM-164 induces cIAP1 degradation and also enhances radiosensitivity [304]. In hepatocellular carcinoma (HCC), combination treatment with SM-164 and doxorubicin markedly enhances caspase-3/-9 activation, promotes PARP cleavage, and suppresses AKT signaling, thereby increasing chemosensitivity to doxorubicin [304]. This chemosensitizing mechanism is associated with downregulation of XIAP, reinforcing the role of SM-164 as a potent IAP antagonist capable of restoring apoptotic signaling and overcoming drug resistance [305].

ASTX660 (Tolinapant), a potent non-peptide monovalent Smac mimetic developed by Astex, binds to the BIR3 domains of cIAP1, cIAP2, and XIAP [306]. In apoptosis-resistant bladder cancer, ASTX660 effectively induces necroptosis, whereas in head and neck squamous cell carcinoma (HNSCC), it displays limited monotherapy efficacy in murine oral cancer and several HNSCC cell lines [307]. Notably, in combination regimens, ASTX660 synergizes with radiotherapy and anti-PD-1 immunotherapy, markedly prolongs survival in MOC1 allograft models, and exhibits synergy with TRAIL/TNFα in both HPV-negative and select HPV-positive HNSCC lines [308]. ASTX660 enhances tumor–immune interactions by sensitizing MOC22 cells to TNFα and MOC1 cells to TNFα, TRAIL, FasL, and cisplatin, increasing MOC1-ova susceptibility to cytotoxic T cells-mediated killing, and augmenting tumor-infiltrating lymphocytes (TILs) cytotoxicity [382]. The combination of TNFα + ASTX660 induces potent immunogenic cell death (ICD), driving CD8^+^ T cell and dendritic cell responses and enhancing antitumor immunity under radiotherapy in both HPV (–) and HPV (+) HNSCC xenografts [308]. Multiple ongoing clinical trials are exploring ASTX660 in advanced malignancies, including ASTX660 + pembrolizumab in immunotherapy-resistant TNBC, cervical cancer, and glioblastoma (NCT05082259) [309], ASTX660 + chemoradiotherapy as neoadjuvant for rectal cancer (NCT05912075) [310], ASTX660 + radiotherapy for cisplatin-ineligible locally advanced HNSCC (NCT05245682) [311], ASTX660 ± bevacizumab + paclitaxel in recurrent ovarian cancer (NCT06393751) [312], ASTX660 ± oral decitabine/cedazuridine in refractory peripheral T-cell lymphoma (NCT05403450) [312], and ASTX660 + eribulin for advanced TNBC (NCT06590558).

APG-1387, a divalent, orally SMAC mimetic designed by Ascentage, binds to cIAP1, cIAP2, and XIAP [313]. Mechanistically, APG-1387 promotes degradation of IAPs, activation of RIPK1 and caspase-8, and induction of TNFα-dependent apoptosis [313]. It also enhances IL-12 secretion and stimulates immune activation, suggesting a dual pro-apoptotic and immunomodulatory role [102]. In hepatocellular carcinoma (HCC), APG-1387 alone effectively reduces IAP protein levels but exerts minimal cytotoxicity in vitro. However, combined treatment with TNFα or TRAIL markedly decreases viability and proliferation in HepG2 and HCCLM3 cells, even with cancer stem-like features, by activating caspase-dependent apoptosis and partially RIPK1-driven necroptosis [271]. These results support the use of APG-1387 in combination with immune checkpoint inhibitors, such as PD-1 antibodies, to overcome resistance in HBV-associated HCC [384]. In ovarian cancer, APG-1387 suppresses IAP expression and facilitates assembly of the caspase-8/FADD/RIP1 complex, driving RIPK1- and TNFα-dependent apoptosis [385]. Its combination with TRAIL induces synergistic cleavage of caspase-3 and PARP, selectively targeting cancer stem cells (CSCs) and reducing tumor stemness [386]. In ovarian and colorectal tumor models, APG-1387 enhances IL-12 secretion from tumor cells, thereby increasing tumor-infiltrating CD3^+^ NK1.1^+^ cells and synergistically augmenting the antitumor efficacy of anti–PD-1 therapy [314]. Likewise, in nasopharyngeal carcinoma (NPC) models (S-18/S-26), APG-1387 triggers degradation of cIAP1/2/XIAP, inhibits cell migration, and enhances chemosensitivity to cisplatin and 5-fluorouracil, with co-treatment with TNFα markedly increasing apoptosis [382]. Beyond conventional cytotoxic regimens, APG-1387 exhibits significant synergy with targeted and immune therapies. Preclinical evidence demonstrates that combining APG-1387 with PARP inhibitors (e.g., olaparib) in BRCA1/2-mutant tumors or with MEK inhibitors (e.g., trametinib) in KRAS-mutant pancreatic cancer enhances apoptosis and improves anti-tumor activity [315]. Clinically, APG-1387 has entered multiple phase I/II studies. A Phase I monotherapy trial in chronic hepatitis B (CHB) patients demonstrated safety and pharmacodynamic activity, followed by an ongoing Phase II trial combining APG-1387 with entecavir (NCT04568265), potentially offering a novel therapeutic avenue for chronic HBV infection [316]. A Phase I combination therapy trial in advanced solid tumor patients demonstrated that APG-1387 was well tolerated with no dose-limiting toxicities, the 45 mg weekly dose combined with toripalimab was selected for Phase II and showed antitumor activity in PD-1/PD-L1–naïve NPC patients (NCT04284488) [317]. Additional studies, including APG-1387 + anti-PD-1 in advanced solid and hematologic malignancies (NCT03386526) [318], and a dose-finding trial of APG-1387 + nab-paclitaxel/gemcitabine in advanced pancreatic cancer (NCT04643405) [319], are under development.

### Antisense oligonucleotides (ASOs)

ASOs, short DNA or single-strand RNA molecules (8–50 nucleotides), exert gene-silencing effects by either recruiting RNase H to degrade mRNA or sterically blocking ribosome assembly to inhibit translation [387]. Early Survivin-targeted ASOs effectively induce apoptosis in human melanoma cell lines, while subsequent studies demonstrated that both synthetic small oligonucleotides and their expression vectors downregulated Survivin at both transcriptional and translational levels, resulting in reducing proliferation, enhancing caspase-dependent apoptosis, and increasing sensitivity to cytotoxic agents (e.g., TRAIL, cisplatin, paclitaxel, imatinib, etoposide) as well as radiation [320]. Similarly, XIAP-targeted ASOs directly promote cell death and sensitize tumor cells to chemotherapy and radiotherapy. In vitro, XIAP ASOs markedly enhanced doxorubicin and paclitaxel cytotoxicity in lung cancer H460 cells [388], while in vivo xenograft studies showed synergistic tumor suppression and growth delay when combined with vinorelbine or radiotherapy [389].

LY2181308, an 18-mer 2-O-methoxyethyl-modified antisense oligonucleotide specifically targeting Survivin mRNA, induces caspase-3–dependent apoptosis, causes G2/M cell-cycle arrest, and promotes multinucleated cell formation in hepatocellular carcinoma models. In SW480 colorectal cancer cells, LY2181308 also enhances radiosensitivity and demonstrates synergistic chemosensitization to gemcitabine, paclitaxel, and docetaxel, underscoring its potential for combination therapy [321]. Despite strong preclinical efficacy across multiple tumor models, clinical outcomes have been modest. Phase I/II studies (NCT00415155, NCT00642018, NCT01107444) reported limited therapeutic responses but confirmed favorable safety and tolerability [320]. Notably, in refractory or relapsed acute myeloid leukemia (AML), the combination of LY2181308 with cytarabine and idarubicin (NCT00620321) produced synergistic antitumor activity without dose-limiting toxicities, highlighting its potential in hematologic malignancies [322].

AEG35156, a second-generation antisense oligonucleotide, selectively suppresses XIAP expression in a dose-dependent manner, thereby inhibiting tumor growth in vitro and in vivo [182]. In the H460 (NSCLC) xenograft model, AEG35156 monotherapy reduced tumor volume by 40%, while its combination with docetaxel achieved a 77% reduction within three weeks, demonstrating strong synergistic antitumor activity with standard chemotherapeutic agents [182]. Clinically, AEG35156 has undergone multiple phase I/II trials evaluating its safety dosing, and combination efficacy with docetaxel (NCT00357747, NCT00372736), sorafenib (NCT00882869), and cytarabine/idarubicin in refractory or relapsed acute myeloid leukemia (NCT00363974) [323].

EZN-3042, a third-generation locked nucleic acid antisense oligonucleotide that specifically binds the stop codon within Survivin exon 4, exhibiting greater potency than earlier-generation analogs such as LY2181308 [320]. It effectively downregulates Survivin and Bcl-2 expression at both mRNA and protein levels, thereby amplifying apoptotic responses [320]. In prostate cancer models, EZN-3042 enhances paclitaxel sensitivity both in vitro and in vivo, where Survivin suppression markedly increases caspase-mediated apoptosis [324]. In the Calu-6 (lung cancer) xenograft model, monotherapy reduces Survivin mRNA expression by approximately 60% and inhibits tumor growth by ~40%, and when combined with paclitaxel, achieves > 80% growth suppression, underscoring its synergistic chemosensitizing potential [324]. Despite its strong preclinical efficacy across multiple tumor types, a Phase I clinical trial in pediatric recurrent acute lymphoblastic leukemia (NCT01186328) was terminated early due to dose-limiting toxicities and adverse drug-drug interactions observed during combination chemotherapy [325].

Ribozymes are small catalytic RNA molecules capable of cleaving specific RNA targets through endonucleolytic activity. Among them, hammerhead ribozymes are the best characterized, containing a conserved catalytic core and three base-paired domains that enable sequence-specific cleavage of RNA after NUH triplets [326]. Compared to siRNA-based strategies, ribozymes offer superior sequence specificity, but their clinical translation remains constrained by misfolding and RNA degradation when vectorized [327]. Preclinical studies have demonstrated the therapeutic potential of Survivin-targeted ribozymes (RZ-1/RZ-2) in breast cancer cells, which induced apoptosis and enhanced chemosensitivity under stress conditions such as serum starvation or etoposide exposure [328]. Similarly, in androgen-independent prostate cancer xenografts, Survivin-directed ribozymes increased cisplatin sensitivity and significantly reduced tumor growth, suggesting a promising strategy for overcoming chemoresistance in survivin-overexpressing malignancies [329].

### Small molecule inhibitors

Small molecule inhibitors are low-molecular-weight organic compounds ( < 1000 Da) with characterized chemical structures that exert therapeutic effects by specifically binding to protein targets such as enzymes and receptors. Through reversible or irreversible modulation of these interactions, they regulate key biological pathways and are extensively applied in the treatment of cancer, and autoimmune, and inflammatory diseases [390].

YM155, a selective Survivin transcriptional repressor, inhibits Survivin expression by targeting its promoter, thereby activating PUMA-mediated and caspase-3-dependent apoptosis [330]. It exhibits broad antitumor activity across multiple cancer cell lines, induces apoptotic responses in hormone-refractory prostate cancer (HRPC) PC-3 cells, and inhibits tumor growth in PC-3 xenograft models [330]. In head and neck squamous cell carcinoma (HNSCC), YM155 downregulates Survivin while concurrently inhibiting the Akt/mTOR pathway and upregulating Beclin-1, leading to enhanced apoptosis and autophagy [331]. Similarly, in esophageal cancer, YM155 induces dose-dependent Survivin suppression and apoptosis [332]. Furthermore, in leukemia cells, YM155 combined with decitabine exhibits synergistic cytotoxicity through upregulation of SLC35F2 and suppression of MCL1 and Survivin expression [333]. Although preclinical efficacy was promising, clinical trials reported limited therapeutic benefit across several solid and hematologic malignancies. Nevertheless, YM155 exhibited favorable safety and tolerability, even in advanced disease settings, though no clinical activity was observed in esophageal cancer [334].

EM-1421 (Terameprocol), a small-molecule transcription inhibitor, targets Sp1-regulated proteins such as Survivin and Cdc2. It activates the mitochondrial apoptosis pathway, induces G2/M cell-cycle arrest, and enhances radiosensitivity in NSCLC [335]. Preclinical studies showed robust tumor suppression across multiple xenograft models. However, clinical outcomes have been mixed: in advanced leukemia, a phase I trial (NCT00664677) reported good tolerability and partial responses in subsets of CML and AML patients [336], whereas a glioma trial (NCT02575794) showed no objective responses, despite 32% disease stabilisation [337]. Furthermore, trials in HPV-associated cervical intraepithelial neoplasia (NCT00154089) and refractory malignancies (NCT00259818) are completed [338].

FL-118, a camptothecin derivative structurally related to irinotecan, acts as a nonselective anticancer agent targeting multiple IAPs. It suppresses Survivin by inhibiting promoter activity and causing DNA damage through topoisomerase I inhibition, while simultaneously downregulating Mcl-1, XIAP, and cIAP2 [335]. Preclinical studies have demonstrated potent antitumor efficacy in head and neck cancer xenografts and colorectal carcinoma, with significant tumor growth inhibition [339].

### Hsp90 inhibitors

Heat shock protein 90 (Hsp90) is a molecular chaperone essential for sustaining proteostasis across numerous client proteins, including Survivin. Its chaperone activity supports post-translational processing and stability of Survivin, a process mediated through Survivin’s BIR domain [19, 391]. Inhibiting Hsp90 disrupts the Hsp90–Survivin complex, leading to Survivin degradation, mitotic defects, and mitochondrial-mediated apoptosis [342]. Therapeutically, targeting the Survivin-HSP90 complex to promote proteasomal degradation of Survivin has emerged as a promising anticancer strategy [392].

Shepherdin, a peptidomimetic derived from Survivin sequences, directly interferes with Hsp90-Survivin interaction. It induces mitochondrial dysfunction and mitotic catastrophe, producing potent apoptotic effects in diverse cancer cell lines and tumor regression in breast and prostate xenografts, while exhibiting minimal cytotoxicity to normal fibroblasts [340, 341].

AICAR (5-Aminoimidazole-4-carboxamide ribonucleotide), a natural purine biosynthesis intermediate, binds to the N-terminal ATP-binding site of Hsp90, triggering ubiquitin-mediated degradation of Survivin and other client proteins [342]. The mechanism drives strong antiproliferative activity in prostate cancer, osteosarcoma, and acute lymphoblastic leukemia, with negligible effects on normal cells [343].

17-DMAG, an HSP90 inhibitor, disrupts the HSP90-Survivin interaction in a time- and concentration-dependent manner, resulting in Survivin suppression, apoptosis induction, and proliferation arrest in hepatocellular carcinoma models [344].

### Cyclin-dependent kinases (CDKs) inhibitors

Flavopiridol and purvalanol A, both CDKs inhibitor, suppress the phosphorylation of Survivin at threonine 34 (Thr34) by the CDK1–cyclin B1 complex during mitosis, a critical mechanism associated with paclitaxel resistance. Inhibition of Thr34 phosphorylation leads to paclitaxel-mediated mitotic arrest and enhanced apoptosis, resulting in potent antitumor efficacy [345].

### Histone deacetylase inhibitors (HDACi)

Dynamic acetylation and deacetylation of histones at lysine residues, which are catalyzed by histone acetyltransferases (HATs) and histone deacetylases (HDACs), regulate gene expression by altering chromatin conformation and accessibility [393]. Aberrant HDAC activity contributes to epigenetic silencing of apoptosis-related genes, including Survivin.

Chlamydocin, a cyclic peptide HDACi inhibitor, induces hyperacetylation of histones H3 and H4, activates caspase-3, and transcriptionally upregulates p21 (CDKN1A), resulting in cell cycle arrest and proteasome-mediated Survivin degradation [346].

Dacinostat (LAQ824), a hydroxamic acid-based HDACi inhibitor, binds to zinc ions in the HDAC catalytic pocket, leading to Survivin downregulation and p21 induction, collectively driving apoptosis in cancer cells [347].

Belinostat (Beleodaq), a pan-HDAC inhibitor, downregulates Survivin expression in a TGF-β dependent manner, causing apoptotic cell death in tumor models [348].

Apigenin, a natural flavonoid with HDAC-inhibitory activity, suppresses all major IAP members including Survivin, and modulates cell cycle genes. In prostate cancer, its chromatin remodeling effects promote growth inhibition and caspase-dependent apoptosis, demonstrating significant therapeutic potential [349].

### Tumor vaccine-based inhibitors

Given that Survivin is abundantly expressed in malignant tumors but nearly absent in normal adult tissues, it is regarded as a potential tumor-associated antigen (TAA) capable of eliciting robust immune responses. This unique tumor specificity positions Survivin as an attractive target for vaccine-based immunotherapy [350]. In preclinical studies, Survivin-derived epitopes have successfully activated cytotoxic CD8^+^ T lymphocytes, producing tumor-suppressive effects across multiple malignancies, including colon cancer, lung cancer, neuroblastoma, and hormone-refractory prostate cancer [351]. In a colon cancer xenograft model, combined treatment with Survivin mRNA and the STAT3 inhibitor stattic significantly increased cell apoptosis, reduced proliferation, enhanced antitumor T cell responses, and improved long-term survival [352]. Clinically, in phase I trials, the HLA-A24-restricted Survivin-2B80-88 epitope vaccine in patients with locally advanced or recurrent OSCC effectively activated CD8^+^ T cells and exhibited acceptable safety, though with modest therapeutic efficacy [353]. Furthermore, synthetic peptide mimetics such as SVN53-67/M57-KLH (DLAQCFFMFKELEGW), presented via MHC class I molecules, elicit strong CD8^+^ T-cell cytotoxicity and systemic immune activation against tumor cells [354]. Subsequent studies confirmed their ability to enhance adoptive immunotherapy by activating dendritic cells, natural killer cells, and cytotoxic T lymphocytes [355].

Additionally, a diverse range of IAP-targeted therapeutic strategies is currently under investigation, including dominant-negative mutants, RNA interference approaches using siRNA and miRNAs, and gene editing technologies with ZFN, TALEN, and CRISPR/Cas systems, as well as oncolytic virus-based therapies designed to selectively eliminate IAP-expressing tumor cells [161].

## Summary and outlook

In summary, IAPs possess E3 ubiquitin ligase activity that regulates cell survival, apoptosis, and necroptosis, modulates innate and adaptive immune responses, and influences cancer development and prognosis. Their frequent overexpression and key roles in cell fate regulation make them attractive therapeutic targets.

Furthermore, leveraging the E3 ubiquitin ligase activity of IAPs, specific non-genetic IAP-based protein erasers (SNIPERs), developed through PROTAC technology, utilize the ubiquitin–proteasome system to degrade target proteins while disrupting their functional interactions with IAPs [317]. This approach enables non-genetic, highly specific, rapid, and reversible knockdown of “undruggable” targets, circumvents the resistance mechanisms associated with conventional inhibitors, and has shown preclinical and early clinical efficacy across multiple malignancies [394, 395].

Although current cancer therapies still rely on multimodal combinations of surgery, radiotherapy, and chemotherapy with limited improvements in OS, IAPs-targeted strategies—ranging from Smac mimetics, and ASOs to related inhibitors and vaccine-based immunotherapy—have shown potent and synergistic anti-tumor effects when combined with conventional or immune-based treatments [396]. Despite significant progress, several translational challenges limit the clinical success of IAP-targeted therapies. First, marked tumor heterogeneity across cancer types results in variable IAP expression patterns and context-dependent functions, complicating the establishment of universal therapeutic strategies. Second, compensatory activation of alternative signaling pathways can induce adaptive resistance and reduce therapeutic efficacy. Combination regimens targeting complementary pathways may help overcome this limitation. Third, adverse effects of IAP inhibition, including cytokine release and inflammation, highlight the need for improved selectivity and optimized delivery systems. Finally, the absence of validated biomarkers for patient selection remains a major barrier to clinical translation. Identifying reliable predictive biomarkers and integrating molecular profiling into clinical trials will be essential for precision application of IAP-based therapeutics [397].

Collectively, addressing these clinical and translational challenges through integrative biomarker-driven precision therapy, rational drug combination strategy, optimized dosing and delivery system, and AI-assisted drug development and therapeutic strategy will accelerate the successful implementation of IAP-targeted therapies in precision oncology [398].

## Data Availability

No datasets were generated or analyzed during the current study.
